# Phosphoproteomes of *Strongylocentrotus purpuratus *shell and tooth matrix: identification of a major acidic sea urchin tooth phosphoprotein, phosphodontin

**DOI:** 10.1186/1477-5956-8-6

**Published:** 2010-02-08

**Authors:** Karlheinz Mann, Albert J Poustka, Matthias Mann

**Affiliations:** 1 Max-Planck-Institut für Biochemie, Abteilung Proteomics und Signaltransduktion, D-82152 Martinsried, Am Klopferspitz 18, Germany; 2 Max-Planck-Institut für Molekulare Genetik, Evolution and Development Group, D-14195 Berlin, Ihnestrasse 73, Germany

## Abstract

**Background:**

Sea urchin is a major model organism for developmental biology and biomineralization research. However, identification of proteins involved in larval skeleton formation and mineralization processes in the embryo and adult, and the molecular characterization of such proteins, has just gained momentum with the sequencing of the *Strongylocentrotus purpuratus *genome and the introduction of high-throughput proteomics into the field.

**Results:**

The present report contains the determination of test (shell) and tooth organic matrix phosphoproteomes. Altogether 34 phosphoproteins were identified in the biomineral organic matrices. Most phosphoproteins were specific for one compartment, only two were identified in both matrices. The sea urchin phosphoproteomes contained several obvious orthologs of mammalian proteins, such as a Src family tyrosine kinase, protein kinase C-delta 1, Dickkopf-1 and other signal transduction components, or nucleobindin. In most cases phosphorylation sites were conserved between sea urchin and mammalian proteins. However, the majority of phosphoproteins had no mammalian counterpart. The most interesting of the sea urchin-specific phosphoproteins, from the perspective of biomineralization research, was an abundant highly phosphorylated and very acidic tooth matrix protein composed of 35 very similar short sequence repeats, a predicted N-terminal secretion signal sequence, and an Asp-rich C-terminal motif, contained in [Glean3:18919].

**Conclusions:**

The 64 phosphorylation sites determined represent the most comprehensive list of experimentally identified sea urchin protein phosphorylation sites at present and are an important addition to the recently analyzed *Strongylocentrotus purpuratus *shell and tooth proteomes. The identified phosphoproteins included a major, highly phosphorylated protein, [Glean3:18919], for which we suggest the name phosphodontin. Although not sequence-related to such highly phosphorylated acidic mammalian dental phosphoproteins as phosphoryn or dentin matrix protein-1, phosphodontin may perform similar functions in the sea urchin tooth. More than half of the detected proteins were not previously identified at the protein level, thus confirming the existence of proteins only known as genomic sequences previously.

## Background

Sea urchin is an important model organism for developmental biology and in particular skeletogenesis, providing insight into common principles of biomineralization [1-3]. Like other biominerals, sea urchin skeleton elements are composite materials containing, in addition to the mineral component, a small percentage of biopolymers, the organic matrix. This network of organic molecules, pervading the mineral, controls the formation of biominerals and contributes to their final properties [4-6]. Research on the sea urchin model was boosted by the recent publication of the complete *Strongylocentrotus purpuratus *genome [7,8]. The genome sequence enabled the search for potential novel biomineralization-related proteins and their transcriptional regulation [9]. It also made possible the direct identification of matrix proteins by mass spectrometry-based proteomics, revealing an unexpected complexity of test (shell), spine and tooth proteomes [10,11]. However, proteomes cannot be considered complete without determination of post-translational modifications. One of the most widespread post-translational modifications occurring in proteins of biominerals is phosphorylation [12]. Organic matrices of biominerals as diverse as mammalian tooth and bone [13], chicken eggshell [14] or mollusk shell [15] contain phosphoproteins. In a few cases, such as crustacean orchestin [16], mammalian phosphoryn [17] or osteopontin [18,19], phosphorylation was shown to be crucial for proper folding, calcium binding, and other function-related properties of these proteins. Phosphorylation of mammalian extracellular proteins involves casein kinase-like enzymes of the endoplasmic reticulum and Golgi apparatus [12,20,21] and membrane-bound ectokinases [22,23]. Although sea urchin is a major model organism for biomineralization studies, data about phosphoproteins in sea urchin skeletal elements are scarce.

At least 353 putative protein kinases were predicted to be encoded in the *S. purpuratus *genome [24]. Some of these kinases apparently play a role in skeleton formation, as shown by kinase inhibition studies in embryos and cultured spicule-producing mesenchymal cells [25-28], where specific kinase inhibitors prevented formation of skeletal elements. However, only one study reported the presence of phosphorylated proteins in a sea urchin mineralized structure, the tooth of *Lytechinus variegatus *[29]. However, no phosphorylation sites were identified. In test (the commonly used name for the sea urchin shell), phosphate groups were reported to be attached to unidentified matrix molecules [30].

Mass spectrometry has become the method of choice for detection of protein phosphorylation during the last decade because the method is highly sensitive, does not need radioactive labeling, enables determination of the modified amino acids, and does not require purification of the proteins [31]. MS-based phosphoproteomics involves the enzymatic cleavage of mixtures of proteins, fractionation of the peptides by nanoscale liquid chromatography, and analysis by mass spectrometry. However, because phosphopeptides usually constitute only a minor fraction of the peptide mixture used in automated high-throughput analysis of complex mixtures, phosphopeptides are usually enriched and specialized mass spectrometric methods, such as neutral loss-dependent MS^n^, have to be used for their detection [32]. The phosphoproteomes of sea urchin test (shell) and tooth organic matrix were analyzed using enrichment of phosphopeptides by reversible adsorption to TiO_2 _and neutral loss-triggered multistage activation [33] in LTQ-Orbitrap and LTQ FT Ultra mass spectrometers. Data were analyzed using MaxQuant [34], a novel integrated suite of algorithms developed for the analysis of high-resolution mass spectrometry data. Using this toolkit, 21 phosphoproteins were identified in test matrix and 15 in tooth matrix.

## Materials and methods

### Preparation of organic matrix

Sea urchins were killed by freezing. The shells (also called tests) were cut into two halves and emptied. The skeleton elements were soaked in sodium hypochlorite solution (6-14% active chlorine_; _Merck, Darmstadt, Germany) for 1 h with four changes of solution and ultrasonic treatment (Branson Sonifier model 1200) for 5 min after every change. The cleaned, disconnected calcified elements were washed with water, air dried, and collected separately. Teeth were powdered with mortar and pestle and the powder was washed with sodium hypochlorite again [11]. The water-washed, air-dried test plates and tooth powder were separately demineralized in 50% acetic acid (20 ml/g of dry biomineral) for 14-16 h at 4-6°C. The turbid suspension was dialyzed successively against 2 × 10 vol. 10% and 2 × 10 vol. 5% acetic acid at 4-6°C (Spectra/Por 6 dialysis membrane, molecular weight cut-off 1000; Spectrum Europe, Breda, The Netherlands). The precipitate, which formed during dialysis, and the clear supernatant were lyophilized together.

### Preparation of peptides and enrichment of phosphopeptides

Test and tooth matrix proteins were carbamidomethylated as before [35] using iodoacetamide instead of iodoacetic acid. Reagents were removed by dialysis against 5% acetic acid. The protein concentration in carbamidomethylated organic matrix was determined by amino acid analysis in a Biotronik LC3000 analyzer after hydrolysis of the sample in 6 M HCl for 24 h at 110°C.

Organic matrices extracted from a total of nine tests were pooled. Aliquots of 2 mg of carbamidomethylated test matrix were suspended in 10 mM Tris buffer, pH 8, containing 6 M urea and 2 M thiourea, and cleaved with lysyl endopeptidase (Wako Chemicals, Neuss, Germany). After 8 h the reaction mixture was diluted with 0.05 M ammonium hydrogen carbonate to 2 M urea/thiourea and trypsin (sequencing grade modified trypsin, Promega, Mannheim, Germany) was added for 14 h of incubation. In a second approach lysyl endoprotease cleavage products of 2 mg test matrix were further cleaved with endoproteinase Asp-N for 14 h in 2 M urea (sequencing grade; Roche, Mannheim, Germany). Finally, cleavage products of 2 mg matrix samples were successively cleaved with lysyl endopeptidase (14 h), trypsin (8 h), and endoprotease Asp-N (14 h). The enzyme to matrix ration (w/w) in proteolytic digests was 1:50 for lysyl endopeptidase, 1:100 for trypsin and 1:300 for endoproteinase Asp-N. Incubations were performed at room temperature. Reactions were stopped by addition of trifluoroacetic acid to pH ~2. Insoluble material was removed by centrifugation and the supernatant was dried by vacuum centrifugation. Each combination of enzymes was applied to three or four 2 mg aliquots.

Tooth matrix extracted from 300 teeth from 60 sea urchins was combined. Aliquots of 1 mg carbamidomethylated matrix were cleaved with combinations of lysyl endopeptidase and trypsin or lysyl endopeptidase (8 h), Asp-N (14 h) and trypsin (8 h), as described for test matrix. Each cleavage method was performed with three aliquots of tooth matrix.

Phosphopeptides were enriched by reversible binding to TiO_2 _beads (GL Sciences, 10 μm beads) in a batch protocol [14,36,37]. Briefly, 2,5-dihydroxybenzoic acid was added to the acidified peptide mixture to a final concentration of 5 mg/ml and the mixture was incubated with 10 mg of washed beads/sample for 3 h at room temperature. The mixture was then briefly centrifuged to sediment the beads and the supernatants were incubated for another 3 h with a fresh batch of beads. The loaded TiO_2 _beads were washed with 50% acetonitrile in 0.1% trifluoroacetic acid, and the bound peptides were eluted with 15% NH_4_OH. The eluted peptide mixtures were acidified to pH~2 with trifluoroacetic acid and cleaned with C_18 _Stage (stop and go extraction) Tips [37].

### LC-MS and data analysis

C_18 _reversed phase LC and mass spectrometric analysis was performed using a Proxeon Easy-nLC (Proxeon Biosystems, Odense, Denmark; software version 2.0) coupled to a LTQ-Orbitrap or LTQ-FT Ultra mass spectrometer (Thermo Fisher Scientific) via a nanoelectrospray ion source (Proxeon Biosystems). Full scans were recorded in the Orbitrap analyzer at a resolution of 60,000 or in the FT-ICR with a resolution of 100,000 (at *m/z *= 400) followed by MS/MS of the ten most intense peptide ions in the LTQ analyzer. Neutral loss-triggered multistage activation (Pseudo MS^n ^[33]) for simultaneous fragmentation of neutral loss product and precursor was enabled at -97.97, -48.99 and -32.66 Th relative to the precursor ion, corresponding to a neutral loss of phosphoric acid from singly, doubly and triply charged ions.

Data analysis was performed using MaxQuant v1.0.12.33 [34]http://www.maxquant.org/, a software package making use of the Mascot search engine (Matrix Science, London, UK; version 2.2.04) for database searches. The database used consisted of the *Strongylocentrotus purpuratus *annotated gene models (Glean3) protein sequence database (ftp://ftp.hgsc.bcm.tmc.edu/pub/data/Spurpuratus/fasta/Annotation ([7]; see also http://goblet.molgen.mpg.de/cgi-bin/seaurchin-genombase.cgi for further information about Glean [38]), the corresponding reversed database, and the sequences of common contaminants including human keratins from IPIhuman (a total of 58052 sequences). Carbamidomethylation was set as fixed modification. Variable modifications were oxidation (M), N-acetyl (protein), pyro-Glu/Gln (N-term) and phospho (STY). The initial peptide mass tolerance was set to 7 ppm and the MS/MS tolerance was set to 0.5 Da. Two missed cleavages were allowed. The minimal length required for a peptide was seven amino acids. The peptide and protein false discovery rates (FDR) were set to 0.01. The maximal posterior error probability (PEP), which is the probability of each peptide to be a false hit considering identification score and peptide length [34], was set to 0.01. At least one MS^2 ^spectrum of each identified peptide was manually validated considering the assignment of major peaks, occurrence of uninterrupted y- or b-ion series of at least 3 consecutive amino acids, preferred cleavages N-terminal to proline bonds and C-terminal to Asp or Glu bonds, the possible presence of a2/b2 ion pairs, the presence of neutral losses from fragments, and mass accuracy. The ProteinProspector MS-Product program http://prospector.ucsf.edu/ was used to calculate the theoretical masses of fragments of identified peptides for manual validation. Localization probability values for phosphorylation sites were derived as described [39]. In addition to kinase motif prediction comprised in MaxQuant, we used NetPhos http://www.cbs.dtu.dk/services/NetPhos/[40], NetPhosK http://www.cbs.dtu.dk/services/NetPhosK/[41], and Phosida http://www.phosida.com[42] for phosphorylation site and kinase motif identification and prediction.

BLAST analysis was performed with the program provided by NCBI http://www.ncbi.nlm.nih.gov/blast and by searching against the non-redundant database for all organisms. FASTA and MPsrch search programs were used as provided by the European Bioinformatics Institute (EBI, http://www.ebi.ac.uk) searching against UniProt Knowledgebase and UniProtKB/Swiss-Prot protein sequence databases. Domains were predicted with NCBI Conserved Domain Search [43] and the MotifScan program of http://www.expasy.org/tools.

## Results and Discussion

### Test (shell) matrix phosphoproteins

The carbamidomethylated matrix of hypochlorite-washed test plates contained 15-20% protein as determined by amino acid analysis (w/w). Not all of the material was soluble in 8 M urea/thiourea, which was used to suspend the matrix before proteolytic degradation. Residual insoluble material was sedimented after proteolysis and contained 10-15% of the total protein by amino acid analysis. These observations agreed with earlier results indicating that a large part of the sea urchin test matrix is not protein [30,44]. The highest number of peptides was obtained by cleavage with lysyl endopeptidase and subsequent treatment with trypsin. The other protease combinations yielded only few new peptides and no typical endoprotease Asp-N-derived peptide (Table 1). Altogether 27 unique phosphopeptides from 21 phosphoproteins were identified. These proteins contained 37 phosphorylation sites, the majority of which was identified with a localization probability p > 0.75 [39] (Table 1; Additional file 1: Test matrix protein phosphorylation sites; Additional file 2: Selected spectra of test matrix phosphopeptides).

**Table 1:** Test matrix phosphopeptides

Glean3_entry	Protein	Peptide		No. of P	Best Motif	Cleavage	Tot. no.^5^
04136^1b,2^	P19 (Q8MUL3; Sp-P19)	K. _33_IEEGQA**S**GEGAGEEGK_48 _.D (Fig. 2)		**1**^3^	-	KR KD	1 2
04867^1a,b^	Similar to SM30 (Sp-SM30-E)	R. _76_QPGFGNPG**T**PGGR_88 _.Q (Fig. 1)		**1**^3^	ERK/MAPK	KR	2
05419^2,4^	Src family tyrosine kinase (B6DS93; Sp-SFK3)	R. _357_IIEDE**Y**IAR_365 _.E (S1.1)		**1**	EGFR	KR	2
08678^2^	Similar to protein kinase C-delta 1 (Sp-Pkcd)	K. _725_VALSPTDTSMLS**S**INQR_741 _.Q (S1.2)		**2**	CK1	KR	1
11637	Hypothetical protein (Leu-rich repeats) (Sp-Lrr/Igr_14)	R. _290_LGNLPI**S**R_297 _.E (S1.3)		**1**	NEK6	KR KRD	2 3
13143	Hypothetical protein; Glu-rich motif (~aa108-154) (Sp-Hypp_2072)	R. _332_LI**T**PTN**SS**DEEEEK_345 _.D (S1.4)		**3**	CAMK2 CK2	KR KRD	3 4
14937	Hypothetical protein; domains: partial Glyco_hydro_30, Ion_trans (Sp-Trpm3)	K. _1217_NFDDG**S**LELK_1226 _.A (S1.5)		**1**	PLK1	KD KRD	3 1
15408	Hypothetical protein; Glu-rich motif (~aa85-339), Ser-rich motif (~aa1075-1113) (Sp-Hypp_3054)	K. _984_QAESSGV**S**P**T**NSVGDVPDVIMVDG NK_1009 _.T (S1.6)		**2**	CK1	KR	1
15870 27123^2^	Similar to ribosomal protein P1/SP-RPLP1 (Sp-Rpip1)	K. _94_DE**S**EE**S**DDDMGFGLFD_109 _-- (S1.7)		**2**	CK1	KR KD	8 1
16285	Hypothetical protein/similar to FLJ00139 protein, partial; domain:LIM2 (~aa50-110); partial overlap with Glean3:16293	R. _204_FNASG**S**PVGSPQLER_218 _.H (S1.8) R. _204_FNASG**S**PVG**S**PQLER_218 _.H (S1.9)		**1** **2**	GSK3 ERK/MAPK	KR KR	1 3
	(Sp-Hypp_126/Sp-Hypp_820)	K. _1057_PLL**S**DGEEVEK_1069 _.E (S1.10)		**1**	CK2	KR KD KRD	8 11 19
		R. _1086_**S**AAL**S**DDDDQSPVPPAPR_1102 _.K (S1.11)		**2**	PKA, CK1	KR	3
18255	Hypothetical protein; domain: LIM (~aa673-724); Glu-rich motif (~aa358-564), Ser-rich motif (~aa595-661) (Sp-Hypp_912)	K. _332_DAVPVVIIESP**S**SDDLK_348 _.S (S1.12)		**1**	CK1	KR KD KRD	5 5 3
18389	Hypothetical protein; K-rich motif (~aa211-264) (Sp-Hypp_2383)	R. _140_VTPLPVITEEL**S**DPEESPR_157 _.N (S1.13)		**1**	CK2	KRD	2
18649^2^	Similar to pleckstrin homology domain containing, family F (with FYVE domain) 2 (Sp-Plekhf2)	R. _204_NAAPLDQD**S**DDDDDDDEDIDAITR_227 _.N (S1.14)		**1**	-	KR	6
18666	Hypothetical protein/similar to Arg/Ser-rich splicing factor 4 (~aa1768-2208) (Sp-Zcchc11)	R. _1947_DDPDAAEALVPGDDL**S**EEK_1965 _.R (S1.15)		**1**	-	KR KD KRD	7 5 1
20139	Hypothetical protein (Sp-Cola2L_2)	R. _295_FVQDD**S**E**S**NEADEDAPR_311 _.Y (S1.16)		**2**	-	KR	40
		R. _295_FVQDD**S**E**S**NEADEDAPRYPLAPQR_318_.N (S1.17)		**2**	-	KR	3
		R. _339_NVAEAAGL**SS**NEVTQVK_355 _.Q (Fig. 3)		**1**	CK2	KR KRD	22 79
		R. _370_QQQPLPF**S**EQQQEYR_384 _.Q (S1.18)		**1**	NEK6	KR	1
20613	Hypothetical protein (Sp-Hypp_2525)	K. _79_AQD**S**IS**S**ITK_88 _.E (S1.19)		**2**	CK1	KD	2
21739^2^	Similar to ribosomal protein P0/P1/P2; also contained in entry 11703 (Sp-Arp)	K. _233_KEE**S**EEE**S**DDDMGFGLFD_250 _-- (S1.20)		**2**	CK1, CK2	KR	3
24991^4^	Hypothetical protein	R. _142_GP**S**LFGR_148 _.I (S1.21)		**1**	PKA/ AKT	KR KRD	9 3
25515^2^	Similar to sodium bicarbonate cotransporter (Sp-Slc4a_10)	K. _1094_LSG**S**PLPTVR_1103 _.N (S1.22)		**1**	NEK6	KD KRD	6 16
26863^2^	Similar to eukaryotic translation initiation factor 3, subunit 8 (Sp-Eif3c)	R. _35_FFL**S**DDDEEETK_46 _.R (S1.23)		**1**	CK2	KR KRD	3 8
27937 11684^2^	Similar to dynein light chain-1 (Sp-Dynlc2_6a/Sp-Dynlc2_4d)	K. _47_DIAS**Y**IKK_54 _.E (S1.24)		**1**	EGFR	KD	1

**^1a,b^**, protein identified previously in test or spine matrix (**1a**) [10] or tooth matrix (**1b**) [11]. ^2^, known phosphoprotein in sea urchin or other eukaryotic species or similar to a known phosphoprotein. ^3^, non-phosphorylated peptide(s) also identified in the present or previous [10,11] studies. ^4^, also in tooth matrix phosphoproteome (Table 2). ^5^, total number of this phosphopeptide with PEP < 0.01. KR, cleavage with lysyl endopeptidase and trypsin; KD, cleavage with lysyl endopeptidase and endoproteinase Asp-N; KRD, successive cleavage with lysyl endopeptidase, trypsin and endoprotease Asp-N. Phosphorylation sites with a localization probability ≥ 0.75 [39] are printed in bold and underlined. Phosphorylation sites with localization probability 0.5-0.75 are underlined. The entries are ordered according to increasing entry number. For scores and other relevant information see Additional file 1 (Additional file 1: Test matrix protein phosphorylation sites). Annotated best identification spectra are shown in Additional file 2 (Additional file 2: Selected spectra of test matrix phosphopeptides); numbering in brackets is the same as in Additional File 2. SpBase http://sugp.caltech.edu/SpBase/ annotations are given in brackets.

Only two of the test matrix phosphoproteins (P19, SM30-E; Table 1) were identified in recent proteomic studies of spine and test matrix [10] or tooth matrix [11]. This indicates that most of the newly identified proteins were minor components in the matrix of hypochlorite-washed test plates and were detected in the present analysis due to the enrichment for phosphopeptides. The low abundance of phosphoproteins may explain the results of a previous study, which failed to identify any phosphorylated protein in test matrix [30]. Only two of the test matrix phosphoproteins were also identified in tooth matrix (Table 1, Table 2).

**Table 2:** Tooth matrix phosphoproteins

Glean3_entry	Protein	Peptide		No. of P	Best Motif	Cleavage	Tot. no.^5^
00164^1a,b^	Similar to SM-30 (Sp-Clect)	R. _204_LSEPGFF**S**FLR_215 _.E (S2.1)		**1**	-	KR	4
02353 ^2^	Similar to nucleobindin 2a/b	K. _149_DGL**S**AGLPALK_159 _.E (S2.2)		**1**	-	KR	1
03345 ^2^	Dickkopf-1 (aa106-339 of A1XR81_STRPU; Sp-Dkk1))	K. _172_NDGDLLGYL**S**AE**S**GEK_187 _.L (S2.3)		**2**	CK1	KR	8
05419 ^2,4^	Src family tyrosine kinase (B6DS93; Sp-SFK3)	R. _357_IIEDE**Y**IAR_365 _.E (Table 1)		**1**	EGFR	KR	1
10288	Similar to pecanex-like protein 1; domain: partial Transmembrane protein 26 (Sp-Tmem26_3)	R. VLGTG**T**LDR .Q (S2.4)		**1**	-	KDR	4
10589^1a^	Hypothetical protein; pI 4.1, Gly-rich, Asp-rich (Sp-Hypp_1925)	K. _245_PIYIPV**S**VPR_254 _.G (S2.5)		**1**^3^	-	KR KDR	8 11
13763^1a^	Similar to Family with sequence similarity 20, member C/Dentin matrix protein 4; domain: DUF1193 (Sp-Fam20c_1)	K. _407_EAMQPYG**S**DLDDDDFDF -- (S2.6)		**1**	-	KR	4
14308	Hypothetical protein; domains: EFh; similarity to reticulocalbin (Sp-Hypp_112)	K. _194_VVILD**S**LEDFDTNK_207 _.D (S2.7)		**1**	-	KR	4
14805	Hypothetical protein; domain: EFh; similarity to multiple coagulation factor deficiency protein 2	K. _117_DM**S**VNQIADAR_128 _.V (S2.8)		**1**	PLK1	KR	4
15125^1a^	Hypothetical protein: domain: galactose-3-O-sulfotransferase (Sp-Gal3st1_6)	K. _87_EDVGGDEE**S**NLA**S**LEGDK_104 _.D (S2.9)		**2**	GSK3, CK1	KR KDR	2 1
15906^1a^	Hypothetical protein (Sp-Hypp_120)	K. _1237_LSVPQG**S**PLLSR_1248 _.C (S2.10)		**1**	GSK3	KR	1
17588^1a^	Hypothetical protein; Val-, Gly-, Pro-, and Ala-rich regions; domains: 2 KAZAL (Sp-Hypp_136)	K. _316_GVAGG**S**EDVGK_327 _.G (S2.11)		**1**	-	KR	4
17590^1a^ 22278^1a^	Hypothetical protein/Similar to serotonin receptor 2B; Val-, Gly-, Pro-, and Ala-rich regions; domains: 1 KAZAL (Sp-Hypp_2346)	R. _492_APSPAAP**S**APR_502 _.A (S2.12) R. _392_APSAPVRPYAPAPAPQAPSGPSD**S**SEK_418 _.Q (S2.13)		**1**^3^ **1**	- CK1 (S**S**)	KR KDR KR KDR	3 4 19 13
18919^1a^	Hypothetical protein/similar to RPGR/ **phosphodontin**; pI 3.9	K. _52(63)_EM**SS**GQVEEPK_62(73)_.E (S2.14)		**1**^3^	-	KR KDR	3 3
	(Sp-Hypp_2410)	K. _74_EMS**S**GEGEEQPK_85 _.E (S2.15)		**1**	CK2 (**S**S)	KR	3
		K. _97(108,119,130,141,152,163,174)_EI**SS**GEGEQPK_107(118,129,140,151,162,173,184)_.E (S2.16)		**1**^3^	CK2 (**S**S)	KDR	2
		K. _185(229)_EI**SS**GEEEQPNEIS**S**GEGEEPK_206(250) _.E (S2.17)		**2**	CK2 (**S**S)	KR KDR	10 13
		K. _218(285)_EIS**S**GEEEQPK_228(295) _.E (S2.18)		**1**^3^	CK2	KR KDR	6 4
		K. _251(296,307)_EIS**S**GEGEEPK_261(306,317) _.E (S2.19)		**1**^3^	CK2 (**S**S)	KR KDR	4 4
		K. _262(318)_EM**SS**GQVEEQPK_273(329)_.E (S2.20)		**1**	-	KR KDR	8 2
		K. _274(331,341)_EM**SS**GEGYQPK_284(340,351) _.E (S2.21)		**1**	CK2 (**S**S)	KR	3
		K. _296_EIS**S**GEGEEPKEIS**S**GEGEEPK_317 _.E (S2.22)		**2**	CK2 (**S**S)	KR KDR	6 1
		K. _363_EV**SS**GEGEQPK_373 _.E (S2.23)		**1**	CK2 (**S**S)	KR	4
		K. _374_EV**SS**GQVEELK_384 _.G (S2.24)		**1**^3^	CK2 (**S**S)	KR KDR	42 8
		K. _385_GM**SS**GEQEEPK_395 _.E (S2.25)		**1**	CK2 (**S**S)	KR KDR	3 1
		K. _396_EM**SS**GEEEQPK_406 _.E (S2.26)		**1**	CK2	KR	4
		K. _407_EMS**S**GEEEEPK_417 _.E (S2.27)		**1**	CK2	KR	5
24991 ^4^	Hypothetical protein	R. _142_GP**S**LFGR_148 _.I (see Table 1 and Additional File 1)		**1**	PKA/ AKT	KR KDR	13 19

**^1a,b^**, protein identified previously in test or spine matrix (**1a**) [10] or tooth matrix (**1b**) [11]. ^2^, known phosphoprotein in sea urchin or other eukaryotic species or similar to a known phosphoprotein. ^3^, non-phosphorylated peptide(s) also identified in the present or previous [10,11] studies. ^4^, also in tooth matrix phosphoproteome (Table 2). ^5^, total number of this phosphopeptide with PEP < 0.01. KR, cleavage with lysyl endopeptidase and trypsin; KD, cleavage with lysyl endopeptidase and endoproteinase Asp-N; KRD, successive cleavage with lysyl endopeptidase, trypsin and endoprotease Asp-N. Phosphorylation sites with a localization probability ≥ 0.75 [39] are printed in bold and underlined. Phosphorylation sites with localization probability 0.5-0.75 are underlined. The entries are ordered according to increasing entry number. For scores and other relevant information see Additional file 1 (Additional file 1: Test matrix protein phosphorylation sites). Annotated best identification spectra are shown in Additional file 2 (Additional file 2: Selected spectra of test matrix phosphopeptides); numbering in brackets is the same as in Additional File 2. SpBase http://sugp.caltech.edu/SpBase/ annotations are given in brackets.

### Test matrix phosphoproteins related to biomineralization

**Figure 1:**
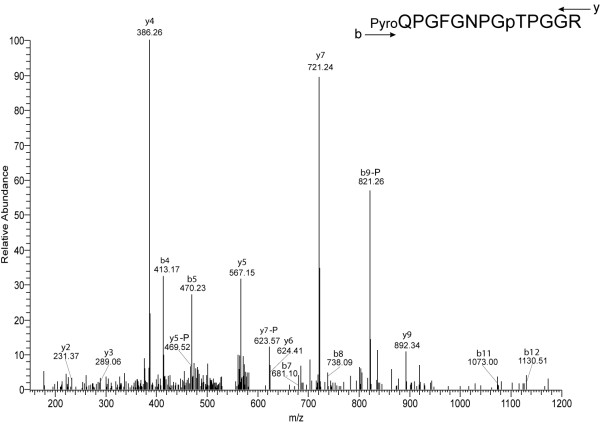
**The phosphorylation site of protein SM30-E**. This peptide was also identified in a non-phosphorylated version in the present and previous studies [10,11], indicating that this site is only partially modified. The spectrum shows an uninterrupted series of y ions (y2-y7). This sequence tag, supplemented by some b ions, and the accurate mass of the complete peptide measured in the orbitrap, allowed the identification of this peptide by database searches. The most intense ions, y7 and y4, are due to preferential cleavage N-terminal of proline residues in position 7 and10 of the peptide sequence. This is a well known feature of Pro-containing peptides frequently used for manual validation of peptide assignments. Loss of H_3_PO_4, _indicated by —P, is first observed in b9 and y5, indicating phosphorylation of Thr in position 9 of the peptide sequence. Loss of NH_3_, indicated by -17, frequently occurs upon fragmentation of Asn-containing peptides. Cyclization of N-terminal Gln to pyroglutamate is common in peptides with N-terminal Gln.

**Figure 2:**
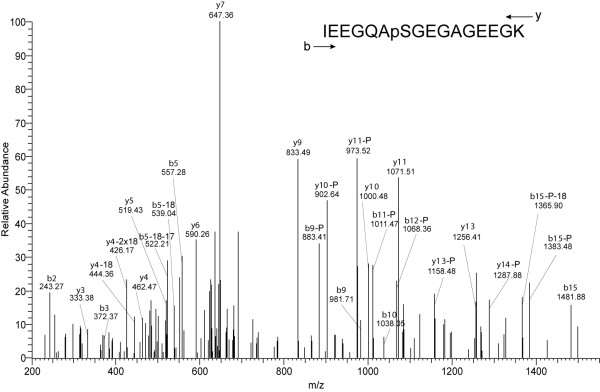
**The phosphorylation site of protein P19**. This peptide was also identified in a non-phosphorylated version in the present and a previous study [11] indicating that the site is only partially modified. P19 was implicated in biomineralization events previously [45] and was identified as a phosphoprotein in *L. variegatus *tooth tissue by phosphor-specific staining [46]. However, previous results also indicated that this protein was at best a very minor component of the intracrystalline matrix [11]. A high match of observed to theoretically expected fragments, including a sequence tag of y3-y7, together with the accurate measurement of the peptide mass, allowed the identification of this peptide. Loss of H_3_PO_4_, indicated by —P, and first observed with y10 indicated the presence of a phospho group at the only serine in the peptide sequence. Loss of NH_3 _and water is indicated by -17 and -18, respectively. These neutral losses are frequently observed in peptides containing Glu and Gln.

The only spicule matrix protein found to be phosphorylated was SM30-E (Table 1, Fig. 1), a major component of the test matrix [10]. However, the analyzed peptide mixture contained more non-phosphorylated copies of this peptide than phosphorylated ones, indicating that the protein was only partially modified at this single site. The test (shell) phosphoproteome (Table 1) also included P19, which was previously tentatively associated with mineralization processes because of its specific expression in spicule-forming primary mesenchymal cells of the sea urchin embryo [45]. P19 was recently identified as a phosphoprotein in *Lytechinus variegatus *teeth, probably as an intracellular component. The phosphorylation sites were, however, not determined [46]. This protein was also detected in the proteome of *S. purpuratus *tooth tissue previously, but was apparently not an intra-crystalline component because it disappeared from the organic matrix proteome after powdering of the teeth and hypochlorite treatment of the powder to remove residual cellular debris and extracellular matrix [11]. The single phosphorylation site, contained in an acidic peptide, was identified from a complex spectrum of product ions resulting from multiple ion activation events (Fig. 2). The site was not completely occupied since non-phosphorylated versions of this peptide were also identified. The powdered tooth matrix phosphoproteome did not contain this protein (Table 2).

### Phosphorylated novel test matrix proteins

**Figure 3:**
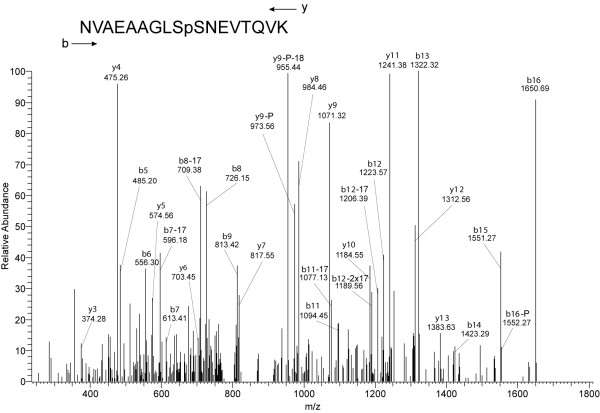
**Identification of the phosphorylation site of peptide NVAEAAGLSSNEVTQVK**. Similar to the spectrum in Fig. 2, this spectrum shows the complexity to be expected from fragmentation of a relatively long peptide by multistage activation. However, the presence of extended series of y and b ions and the accurate mass of the intact peptide measured in the orbitrap enabled the unequivocal identification of the sequence. The phosphorylation site is identified by the increase of 80Da in the y-ion series starting with y8 and the absence of such an increase in b-ions up to b9. Loss of H_3_PO_4_, H_2_O, and NH_3 _is indicated by -P, -18, and -17, respectively. The neutral loss of water and NH_3 _is frequently observed upon fragmentation of peptides containing Asn, Gln, Glu, Ser and Thr, also contained in this peptide.

**Figure 4:**
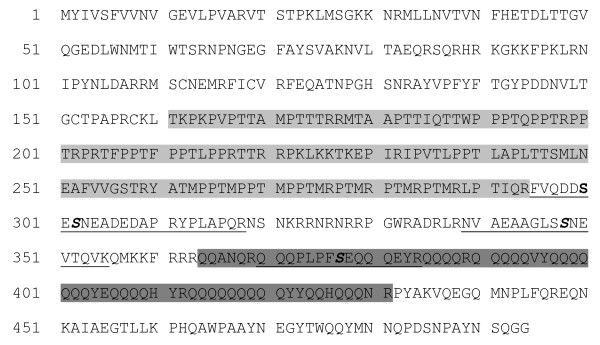
**Analysis of the [Glean3:20139] sequence**. The Thr- and Pro-rich sequence (25% Thr, 26% Pro) is shaded light grey and the Gln-rich sequence (63% Gln) is shaded dark grey. Identified phosphopeptides are underlined, phosphorylated Ser are in bold italics. A phosphorylated region comprising three phospho sites is sandwiched between these two domains, while one phospho site is in the N-terminal region of the Gln-rich domain.

High-quality spectra were obtained for peptides derived from a novel protein (hypothetical protein [Glean3:20139]) [Fig. 3 and Additional File 2: Figs. S1.16, S1.17 and S1.18]. One phosphorylated region, comprising three phosphorylation sites, was sandwiched between an extended proline- and threonine-rich motif and a 70aa-long glutamine-rich motif (Fig. 4). Another phosphopeptide was found in the N-terminal part of the Glu-rich motif (Fig. 4). No known domain signatures or similarities were identified for this sequence in database searches. Another hypothetical protein with multiple phosphorylation was encoded by [Glean3:16285/16293] (Table 1). The 1285 amino acid-long, moderately acidic (calculated pI 5.2) sequence contained a LIM domain (aa50-110), two proline-rich motifs (aa145-186, aa773-789) and a bipartite nuclear localization signal (aa773-789).

### Test matrix phosphoproteins with high similarity to vertebrate proteins with known function or activities

**Figure 5:**
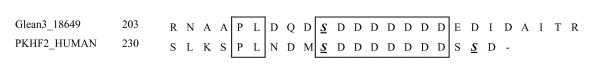
**Alignment of [Glean3:18649] (Similar to pleckstrin homology domain-containing protein) phosphopeptide to its human counterpart**. Phosphoserine 239 [39,47] of the human pleckstrin homology domain-containing family F member 2 protein is also phosphorylated in the homologous sea urchin protein. The phosphorylated serine 248 of human PKHF2 [47] is not conserved in the sea urchin protein.

The test matrix contained several phosphoproteins with known counterparts in vertebrates (Table 1). In many cases the phosphorylation sites of the sea urchin proteins were equivalent to those of mammalian proteins. This is illustrated for a sea urchin protein similar to pleckstrin homology domain-containing F2 [Glean3:18649] in Fig. 5. The human counterpart, PKHF2_HUMAN, has an overall sequence identity of ~63% to the sea urchin protein and was reported to be phosphorylated at Ser239 [39] and 248 [47]. While Ser239 was conserved and phosphorylated in the sea urchin protein, Ser 248 of the human protein was not present in the sea urchin protein. Another test matrix protein containing a conserved phosphorylation site was the src family kinase SFK3 [Glean3:05419]. The phosphorylated Tyr362 of this kinase is equivalent to phosphorylated Tyr426 of human proto-oncogene tyrosine-protein kinase YES (YES_HUMAN, Uniprot: P07947), which has an overall sequence identity of ~60% to the sea urchin protein. This tyrosine is a target of autophosphorylation in mammalian c-YES kinase [48]. The members of the *S. purpuratus *Src family have been cloned recently and their recombinantly produced SH2 domains were shown to be necessary for Ca^2+ ^release at egg fertilization [49]. Also included in this group of proteins with conserved phosphorylation sites were two proteins similar to acidic ribosomal proteins P0/P1/P2 ([Glean3:15870/27123] and [Glean3:21739]). Both peptides were from the acidic C-terminus of these proteins which contains two phosphoserines as part of casein kinase target sites [50]. Entry [Glean3:08678] encoded a protein with similarity to vertebrate protein kinase C-delta 1. The identified phosphopeptide derived from the sea urchin protein matches a region of human protein kinase C type delta (KPCD_HUMAN, [Uniprot:Q05655]), which contains several known phosphorylation sites [51]. Thus, phosphorylated Ser736 of the sea urchin protein is equivalent to phosphorylated Ser654 of the human protein. However, we could not unequivocally localize the second phosphorylated residue of this peptide (Table 1). Finally, phosphorylated Ser38 of the sea urchin protein similar to translation initiation factor 3, subunit 8, [Glean3:26863] was equivalent to phosphorylated Ser39 of the human protein [SwissProt: Q99613] [52].

### Tooth matrix phosphoproteins

Amino acid analysis of carbamidomethylated tooth matrix indicated a protein content of 30-40% (w/w). The tooth phosphoproteome isolated from this matrix comprised 15 proteins (Table 2; Additional file 3: Tooth matrix protein phosphorylation sites; Additional file 4: Selected spectra of tooth matrix protein phosphopeptides), eight of which were already identified previously in proteomic analyses of sea urchin skeletal elements [10,11] by means of non-phosphorylated peptides. These included proteins encoded in entries [Glean3:17588] and [Glean3:17590/22278], which belonged to the group of tooth matrix proteins with Ala- and Pro-rich and acidic Gly-rich motifs described previously [11], and [Glean3:13763], which contained a sequence similar to dentin matrix protein-4(DMP-4)/FAM20C, with up to 50% identity to vertebrate proteins. The acidic C-terminal peptide (Table 2) leading to the identification of this protein was, however, not part of the sequence region of [Glean3:13763] matching to FAM20C/DMP-4 proteins. DMP-4 is a secreted calcium-binding protein abundantly present in dentin and bone [53]. The mouse protein was also reported to be phosphorylated, but at a different site (Phosida database, http://www.phosida.com/). The protein was previously tentatively identified in sea urchin tooth matrix with a different, non-phosphorylated, peptide [11] located in the DMP-matching region, but sequence conservation at that particular site was low. The only protein possibly belonging to the SM30 family and found to be phosphorylated was a protein "similar to SM30" [Glean3:00164] (Table 2). This protein was previously tentatively identified in tooth matrix as a minor protein, but was, to the best of our knowledge, not previously mentioned by others as a spicule matrix protein [9]. Only two of the 15 tooth phosphoproteins were also contained in the test phosphoproteome (Tables 1 and 2).

### Phosphodontin, the major acidic phosphoprotein of tooth matrix

**Figure 6:**
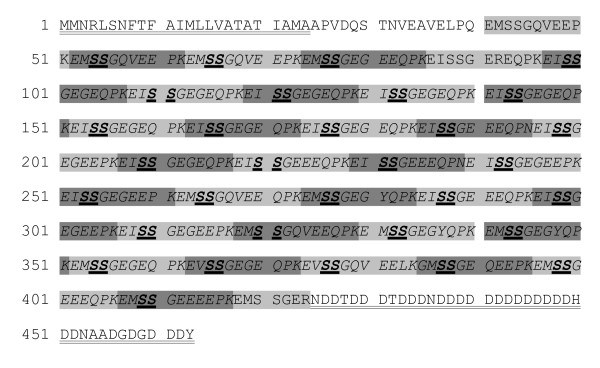
**The sequence of phosphodontin ([Glean3:18919]), the major phosphoprotein of tooth matrix**. The predicted signal sequence and the Asp-rich (72% Asp) C-terminus are doubly underlined. Alternating sequence repeats are shaded light and dark grey. Possible phosphorylation sites derived from experimental data are printed in bold and underlined. Because several repeats occur in multiple identical copies and also non-phosphorylated copies were detected for several of them, it was not possible to determine the extent of phosphorylation of this protein. Furthermore, obviously only one Ser of each repeat can be phosphorylated at a time. Experimental data favor the second Ser of each repeat as the phosphorylation site, but do not completely exclude modification of the first one. Peptides sequenced by MS/MS are printed in italics.

The most interesting phosphoprotein, from the point of view of biomineralization, was encoded as a hypothetical protein in entry [Glean3:18919] and yielded 14 unique phosphopeptides. This protein, for which we propose the name phosphodontin, was already previously identified in a proteomic survey of the tooth matrix by means of non-phosphorylated peptides [11], but in that study it appeared as a minor component. In retrospect it is likely that the concentration of this protein was considerably underestimated before, because most of the possible tryptic peptides were phosphorylated. Phosphorylated peptides were, however, not searched for in the previous study and were therefore not included in the calculation of the exponentially modified protein abundance index (emPAI) used as quantification method [11]. The protein sequence of phosphodontin contained a predicted secretion signal sequence (Fig. 6) which was followed by a region composed of thirty-five 11-12 amino acid-long repeats of various modifications of the sequence EISSGEGEQPK. Most repeats coincided with single tryptic peptides, but in two cases the lysine was substituted by Asn, creating tryptic peptides containing two repeats. Another repeat was interrupted by an Arg (aa86-96), and still another one was part of a longer peptide (aa25-51). The most C-terminal repeat was truncated. These three latter peptides were not identified by MS/MS. The high percentage of Glu (25%) in the sequence and a C-terminal domain consisting almost entirely of aspartic acid conferred to this protein a theoretical pI of 3.9, which would be further lowered by phosphorylation. The modified site in each phosphorylated repeat was one of the two serines in position 3 and 4. In general, the evidence (Additional File 3: Tooth matrix protein phosphorylation sites; Additional File 4: Selected spectra of tooth matrix phosphopeptides) favored the second Ser in each repeat as phosphorylation site. However, we cannot exclude that the first Ser was phosphorylated in peptide variants of the same sequence and composition. While the first serine of most repeats would be classified as part of a CK2 target site, the second serine is in most cases not predicted to be part of any kinase target site by MaxQuant (Table 2). The determination of phosphorylation sites was complicated by the frequent presence of Glu as N-terminal amino acid and Met adjacent to the phosphorylation site in many peptides. Glu frequently occurred either in linear form or as pyroGlu, while Met was present unmodified or oxidized. A few peptides were also identified in non-phosphorylated versions in this (Table 2) or a previous study [11]. With the present set of data we cannot determine the total number of phosphates per protein molecule. This is because several repeats occur more than once in the sequence, giving rise to identical peptides. Thus, for instance, repeat EISSGEGGEQPK was contained eight times in the sequence of [Glean3:18919]. However, the number of identified peptides did not correlate to this high frequency (Tab. 2). Peptides arising from this repeat were also identified in unmodified form in this and a previous study [11]. Therefore it is not clear at present how many, and which, of these identical repeats contained occupied phosphorylation sites. The acidity and the high degree of phosphorylation of the protein is reminiscent of some mammalian tooth matrix phosphoproteins, such as dentin phosphoryn, dentin sialoprotein, or dentin matrix protein-1 [12], but there is no obvious sequence similarity of phosphodontin to any of these mammalian proteins. The lack of predicted structure indicated that this novel tooth matrix phosphoprotein belongs to the growing group of biomineral matrix proteins without a defined structure in the absence of a ligand [54]. Such intrinsically disordered proteins were shown to be frequent targets of kinases and the phosphorylation sites were less well conserved than phosphorylation sites in well-structured sequence regions of known function [55].

### Novel tooth matrix phosphoproteins

Tooth matrix phosphoproteins not previously detected in sea urchin skeletal elements or the test matrix phosphoproteome included a protein similar to nucleobindin 2a/b (Glean3_02353), dickkopf protein-1 (dkk1; [Glean3:03345], a protein with similarity to pecanex-like protein 1/transmembrane protein 26 [Glean3:10288], a protein similar to reticulocalbin [Glean3:14308] and a protein with similarity to multiple coagulation factor deficiency protein 1 [Glean3:14805]. These proteins were probably minor components of the tooth matrix, which became detectable after depletion of major non-phosphorylated proteins.

Vertebrate nucleobindins are calcium-binding phosphorylated proteins of the nuclear envelope and the endoplasmatic reticulum, but secreted forms were reported to occur in bone matrix and tooth matrix [56,57]. The sea urchin protein sequence of [Glean3:02353] was, however, only half the length of vertebrate nucleobindins and contained only one predicted EF hand motif instead of two. Sequence identity to vertebrate proteins was approximately 30% in an overlapping region, but the phosphorylation site was not part of the overlap.

Entry [Glean3:03345] contained amino acids 106-339 of Uniprot entry [A1XR81:STRPU], likely representing Dickkopf protein (Dkk)-1. Dickkopf proteins are secreted inhibitors of the Wnt signaling pathway, which was also implicated in sea urchin development [38]. Human Dkk-1 [39], mouse Dkk-3 [58,59] and chicken eggshell Dkk-3 [14] were reported previously to be phosphorylated at positions found in the same sequence region as the phosphorylation sites of the presumed sea urchin Dkk-1.

Glean3_14308 encoded a sequence with similarity to vertebrate reticulocalbin/calumenin, calcium-binding, EF-hand-containing residents of the ER and Golgi apparatus. The sequence identity of the sea urchin protein to the vertebrate proteins was approximately 30%. However, the sea urchin protein sequence contained a predicted secretory signal sequence and no ER retention signal, such as a C-terminal HDEL, indicating that it was a secreted member of this functionally diverse family of proteins [60].

## Concluding remarks

Using state-of-the-art proteomics instrumentation and software we have detected 21 phosphoproteins in sea urchin test matrix and 15 in tooth matrix. Considering the overlap of two phosphoproteins between these two compartments, we have identified 34 phosphoproteins with 53 unique phosphopeptides containing 64 phosphorylation sites, most of which could be attributed to a single amino acid. Twenty-four phosphorylation sites were not part of a known kinase target site (Tab. 1 and 2). Most of the assigned kinase target sites were casein kinase sites, in agreement with earlier reports indicating that casein kinase-like proteins were largely responsible for the phosphorylation of extracellular proteins. All other assigned sites were part of well-known kinase target sequences, such as EGFR, NEK6 or PKA sites (Tab. 1 and 2). This was in accordance with a recent report predicting almost the same set of kinases as in humans from *in silico *analysis of the *S. purpuratus *genome [24].

Test matrix contained 21 phosphoproteins, only two of which were previously identified as matrix components by means of non-phosphorylated peptides (Table 1). This indicated that all other phosphoproteins were less abundant than the low abundance proteins of the previous proteomic analysis of test matrix [10]. Probably peptides became analyzable only due to the specific enrichment of phosphopeptides. Such minor components likely do not play a role as structural elements but may play a role in signal transduction chains or processing of bulk matrix components. In contrast, eight of the 15 tooth matrix phosphoproteins were previously identified as tooth matrix proteins by means of non-phosphorylated peptides (Table 2). One of them, encoded in entry [Glean3:18919], most probably belongs to the major tooth matrix proteins. In a recent proteomic survey this protein was calculated to be of low abundance only. However, the present study indicated that most of the possible peptides were not taken into account in our previous proteomic survey [11] because the search for phosphorylated peptides was not included. The number of identified and accepted phosphopeptides indicated that this protein, for which we propose the name phosphodontin, would most probably have occupied position two in the abundance ranking [11]. To further characterize phosphodontin it will be necessary to isolate it from tooth matrix in sufficient amounts. Phosphodontin did not show sequence similarity to the better characterized mammalian tooth phosphoproteins. However, properties such as the high degree of phosphorylation and high percentage of acidic amino acids may indicate that this protein has similar functions and importance as the mammalian dental phosphoproteins, and may therefore be an interesting target for future research.

## Abbreviations

FDR: false discovery rate; PEP: posterior error probability.

## Competing interests

The authors declare that they have no competing interests.

## Authors' contributions

KM conceived the study, performed organic matrix and peptide isolation and data acquisition. AJP provided the animals. KM and AJP did database searches and annotations. MM supplied methodological expertise. All authors took part in the design of the study and were critically involved in data interpretation and manuscript drafting. All authors read and approved the final manuscript.

## Supplementary Material

Additional file 1 Test matrix protein phosphorylation sites (xls-file). List of scores, phosphorylation site probabilities, possible kinase target motifs, charge, mass, mass errors and intensity of peptides from test matrix proteins as provided by MaxQuant identification and evaluation software. Click here for file

Additional file 2 Selected spectra of test matrix phosphopeptides (docx-file containing embedded eps-files). For each of the unique peptides one spectrum is supplied. Spectra were saved directly from raw-files and annotated manually using and extending annotations provided by MaxQuant. Click here for file

Additional file 3 Tooth matrix protein phosphorylation sites (xls-file). List of scores, phosphorylation site probabilities, possible kinase target motifs, charge, mass, mass errors and intensity of peptides from tooth matrix proteins as provided by MaxQuant identification and evaluation software. Click here for file

Additional file 4 Selected spectra of tooth matrix phosphopeptides (docx-file containing embedded eps-files). For each of the unique peptides one spectrum is supplied. Spectra were saved directly from raw-files and annotated manually using and extending annotations provided by MaxQuant. Click here for file
