# Supplementary material for: Phosphoproteomes of Strongylocentrotus purpuratus shell and tooth matrix: identification of a major acidic sea urchin tooth phosphoprotein, phosphodontin
Source: Proteome Sci. 2010 Feb 8;8:6. doi: 10.1186/1477-5956-8-6 (PMC2830187; doi:10.1186/1477-5956-8-6)
Supplement: Additional file 2 — Selected spectra of test matrix phosphopeptides (docx-file containing embedded eps-files). For each of the unique peptides one spectrum is supplied. Spectra were saved directly from raw-files and annotated manually using and extending annotations provided by MaxQuant. [file 1477-5956-8-6-S2.DOCX]

**Additional file 2: Selected spectra of test (shell) matrix phosphopeptides.**


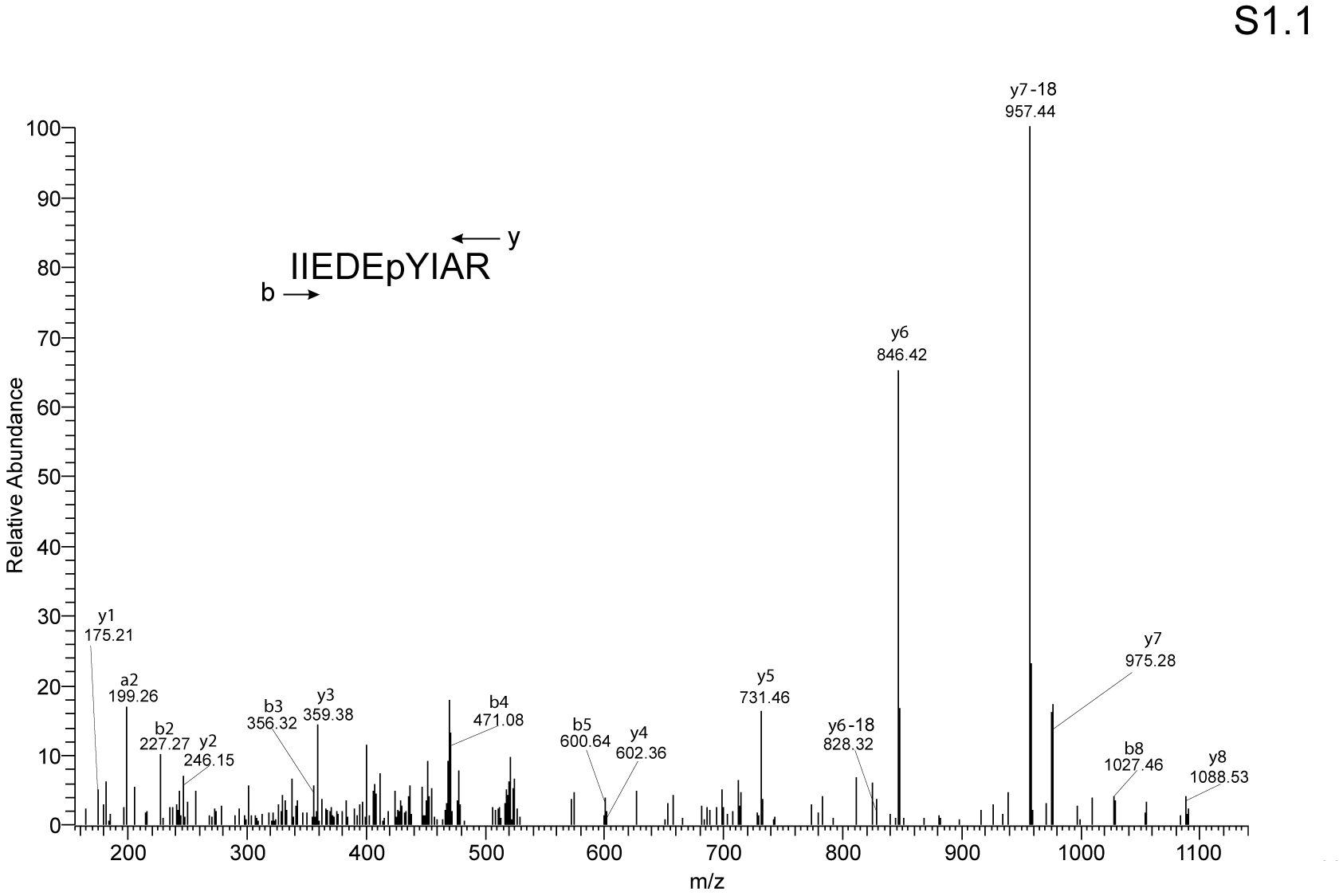


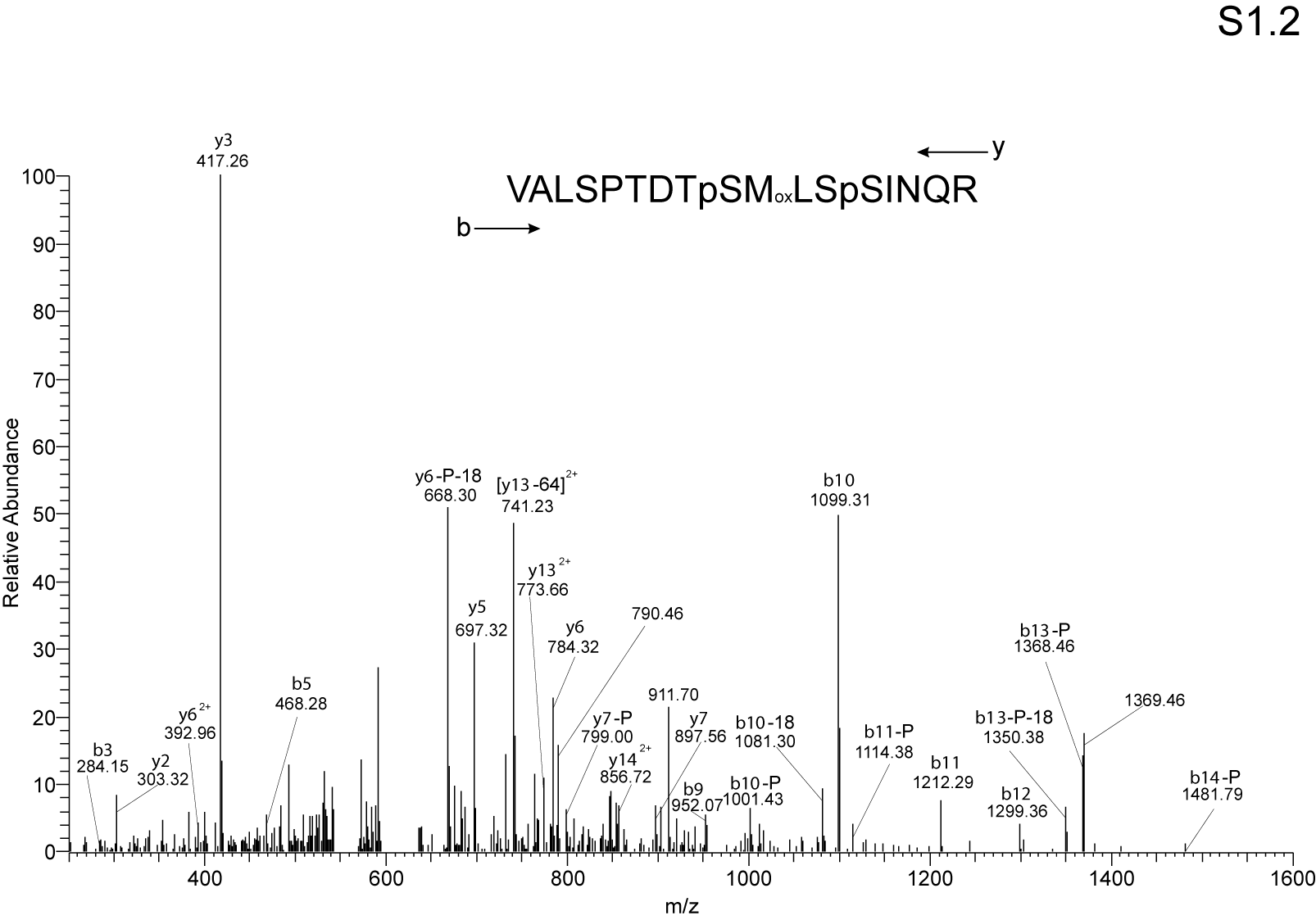


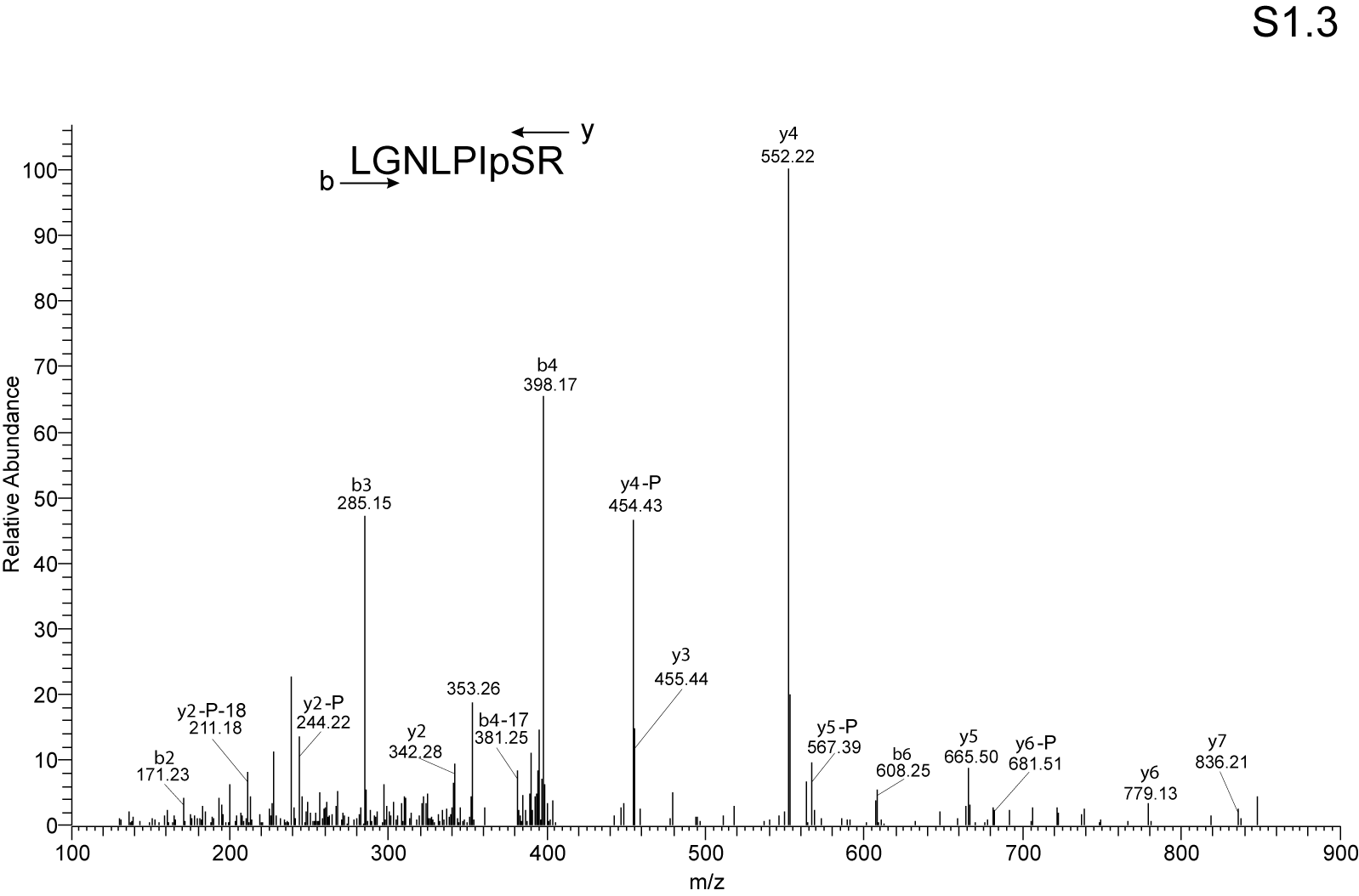


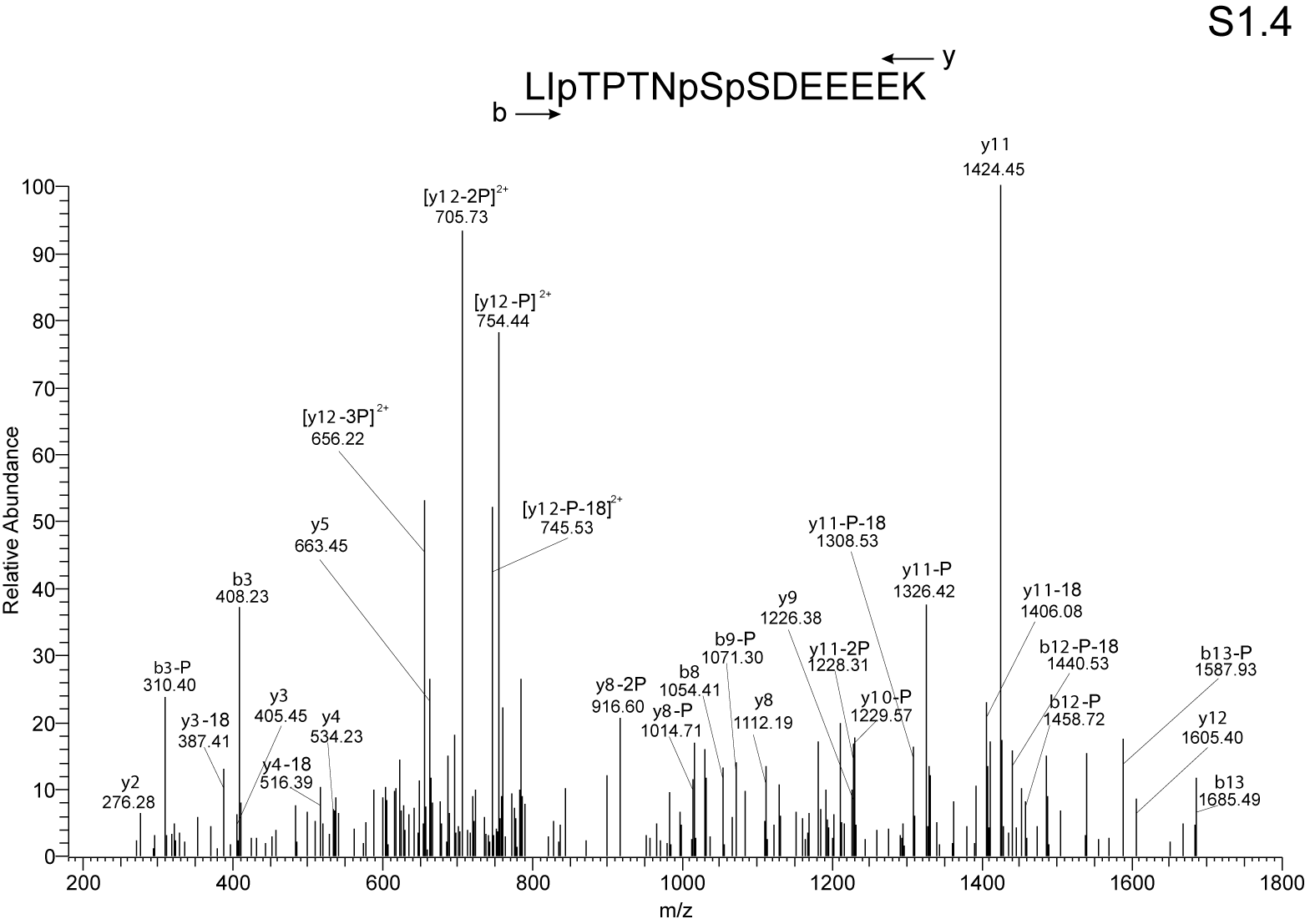


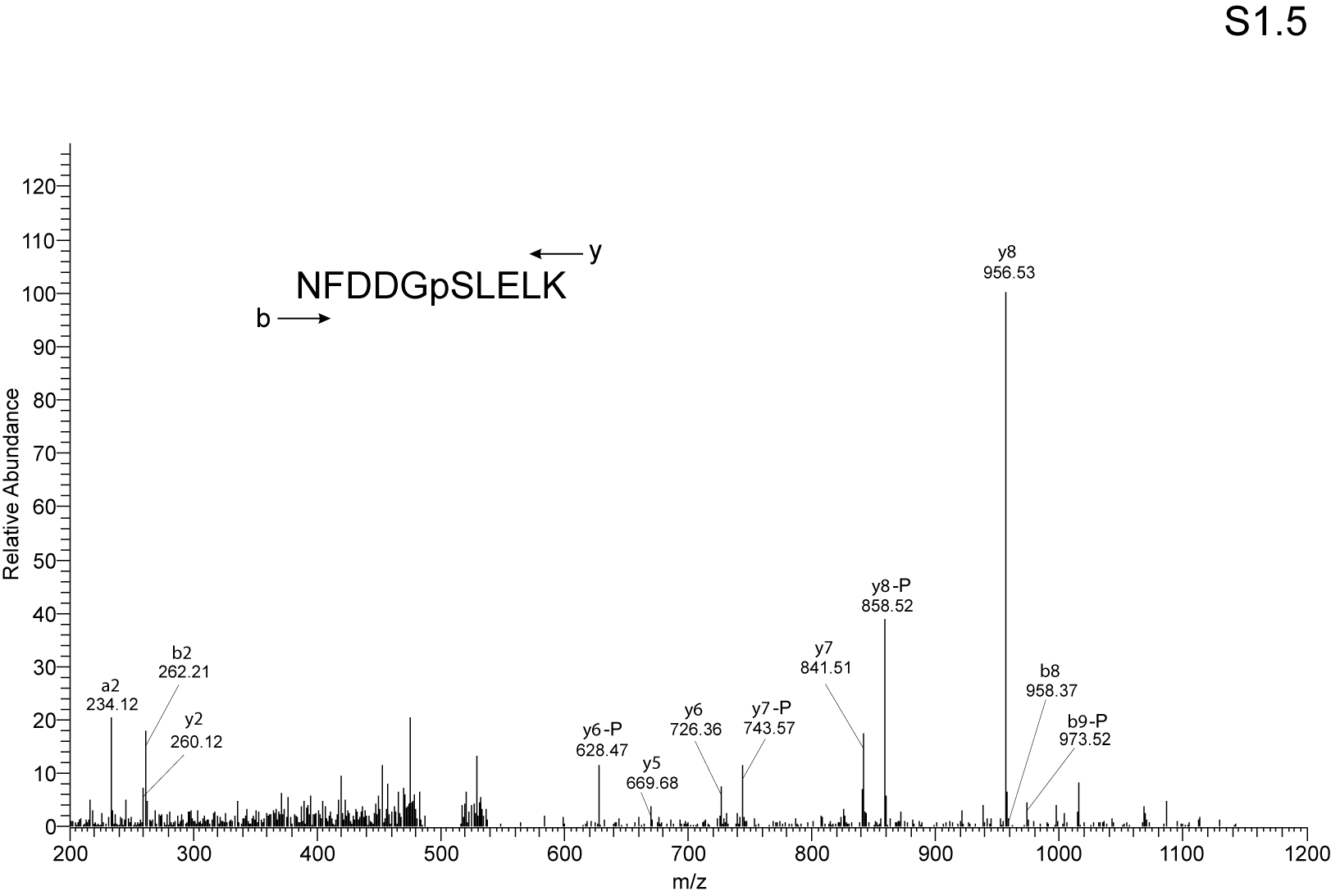


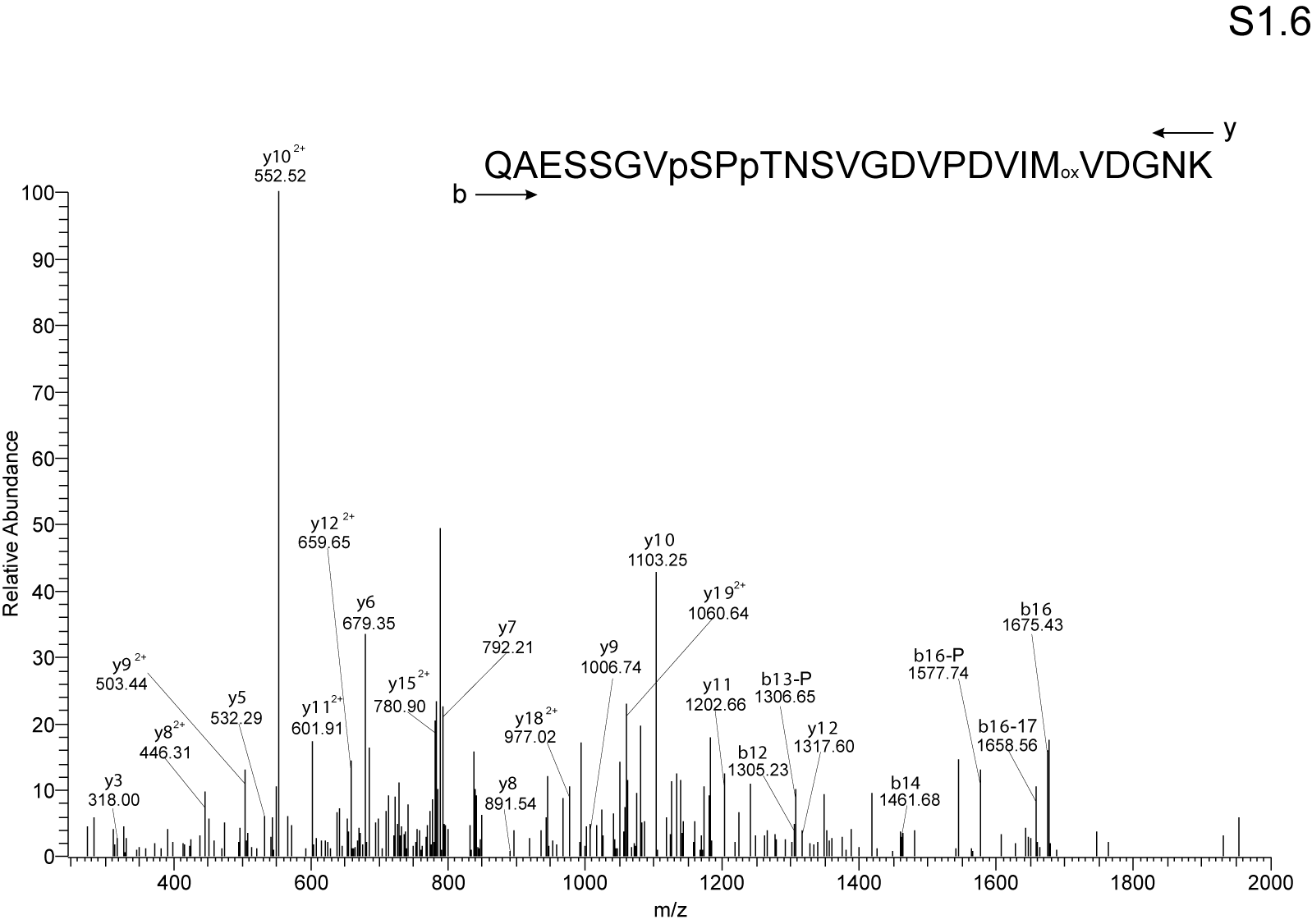


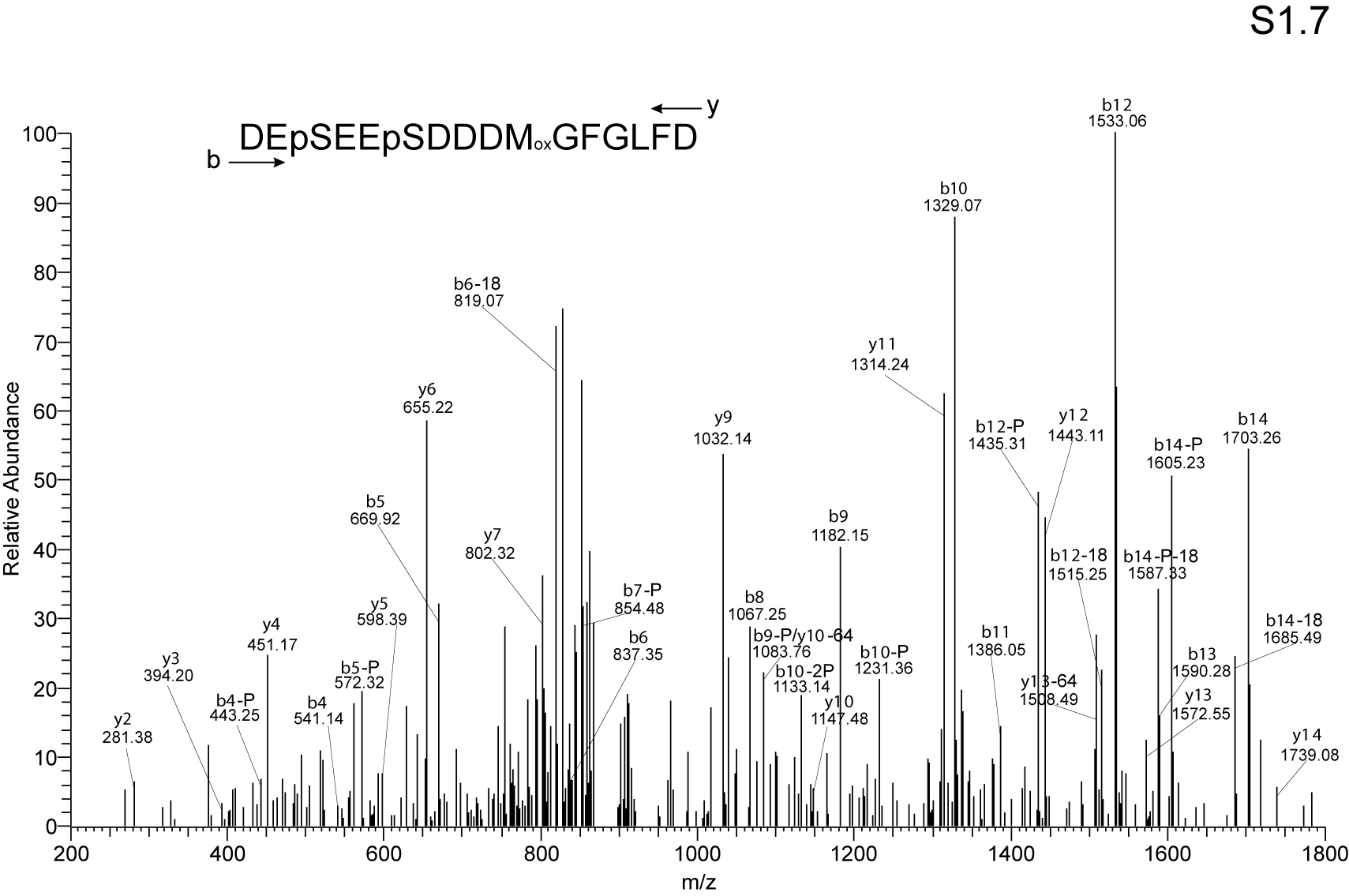


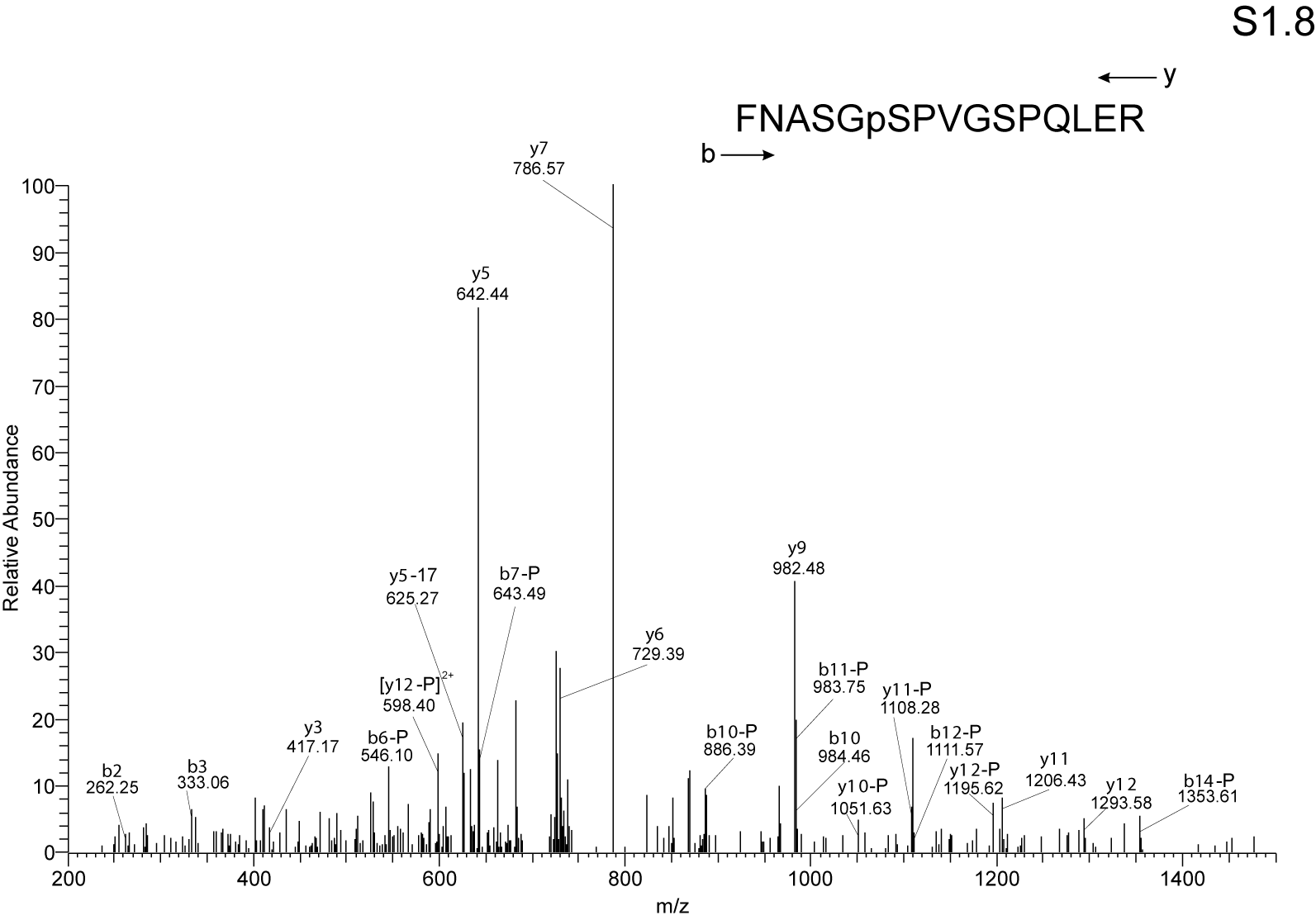


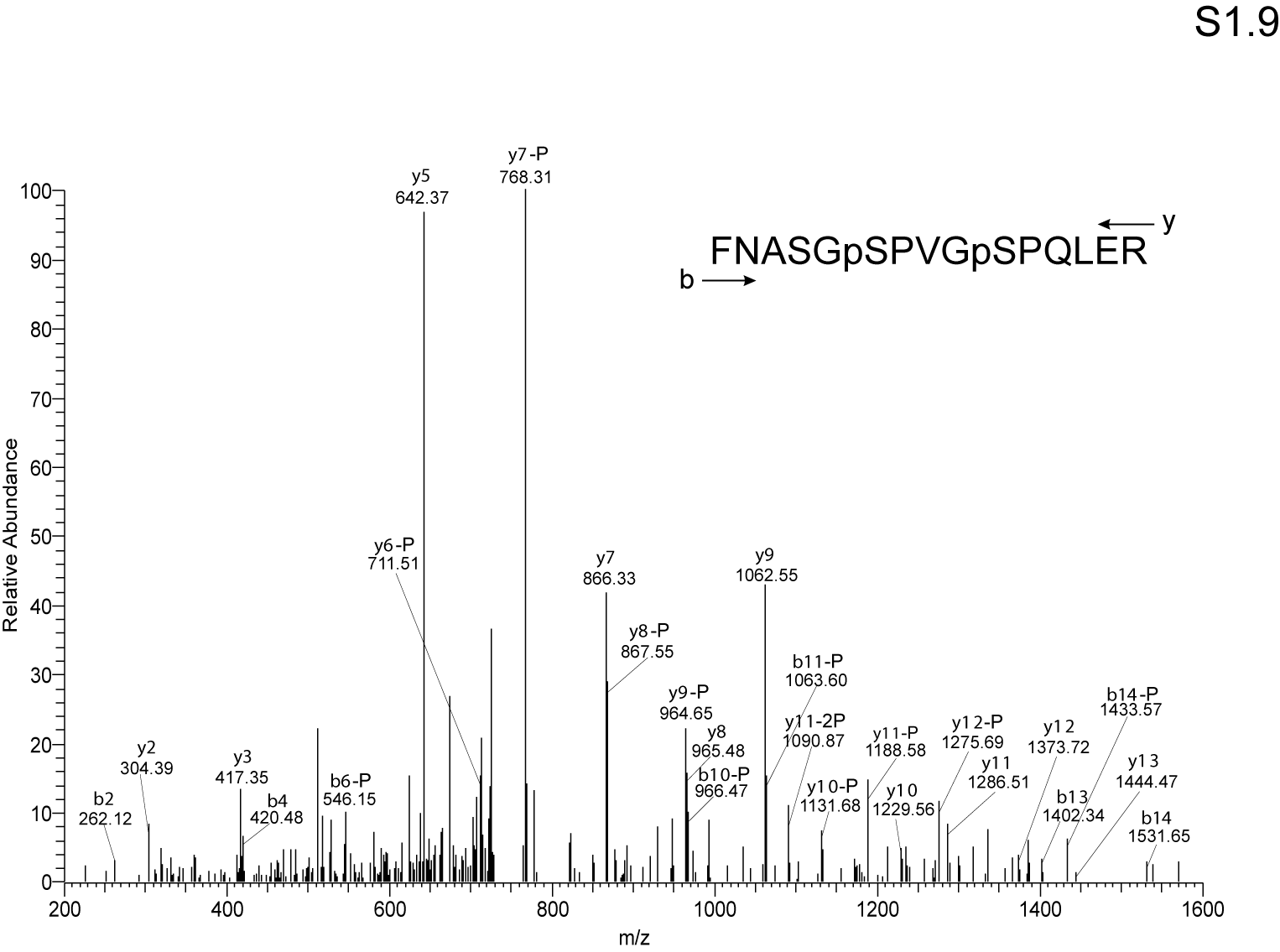


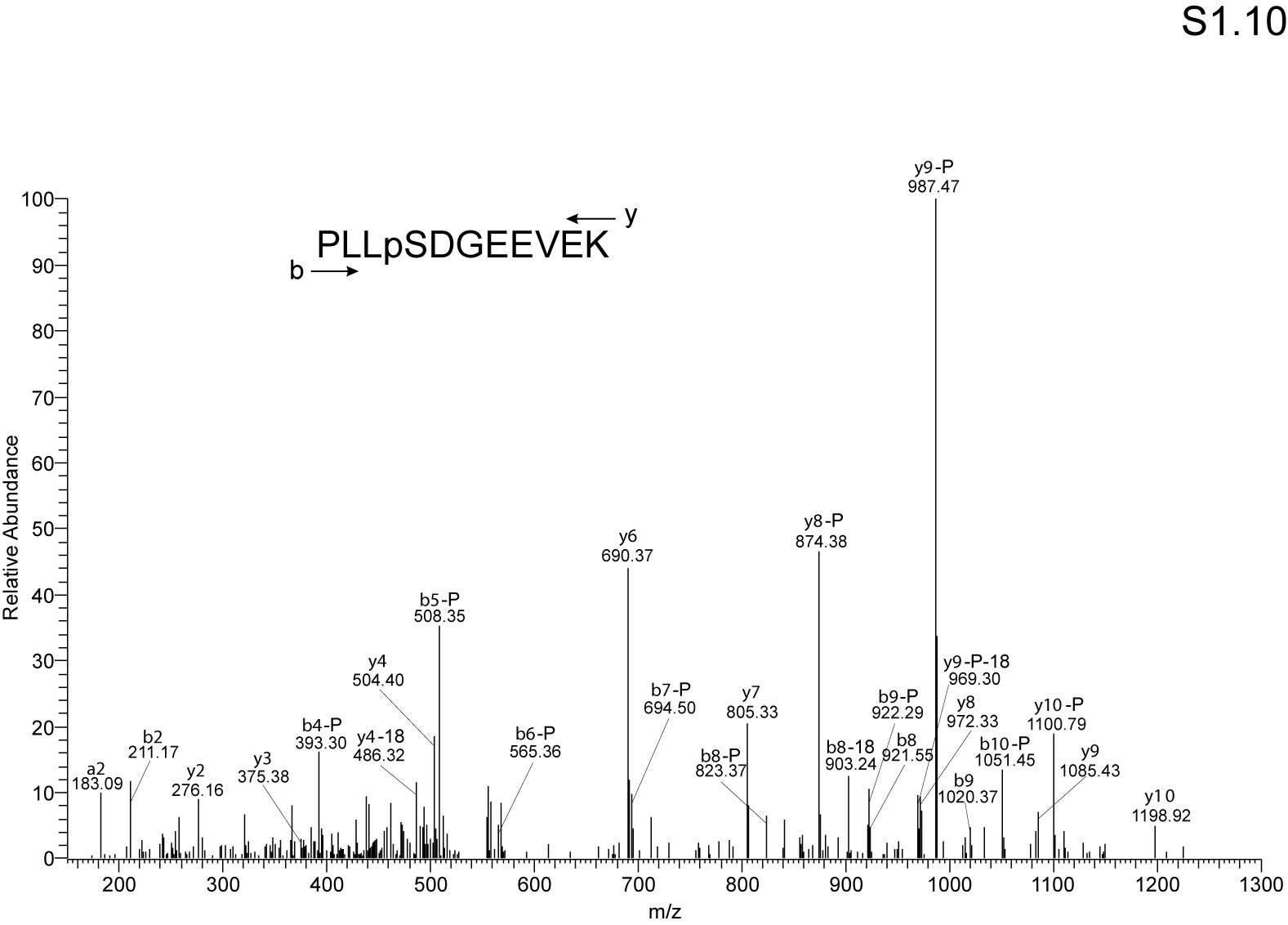


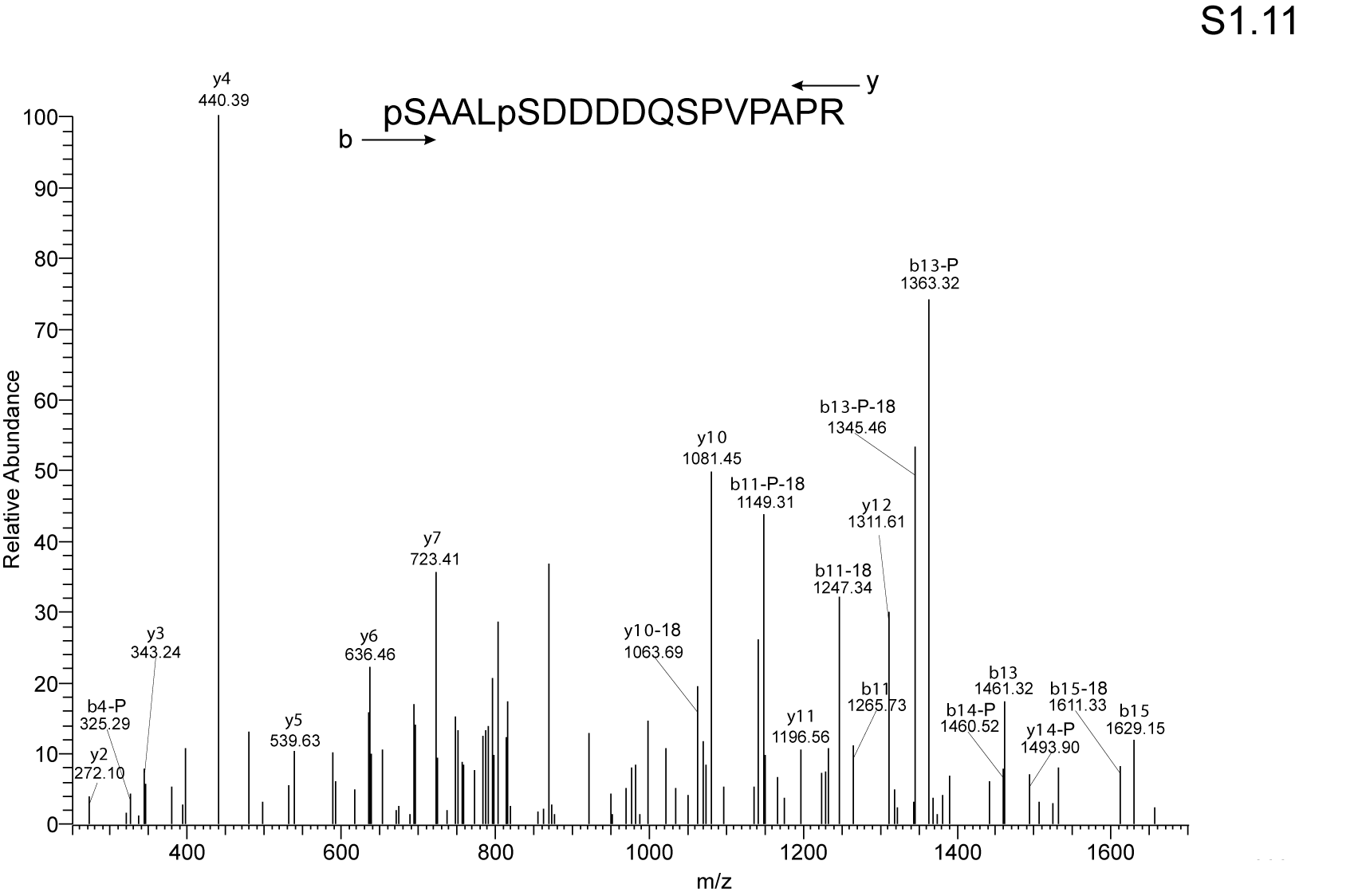


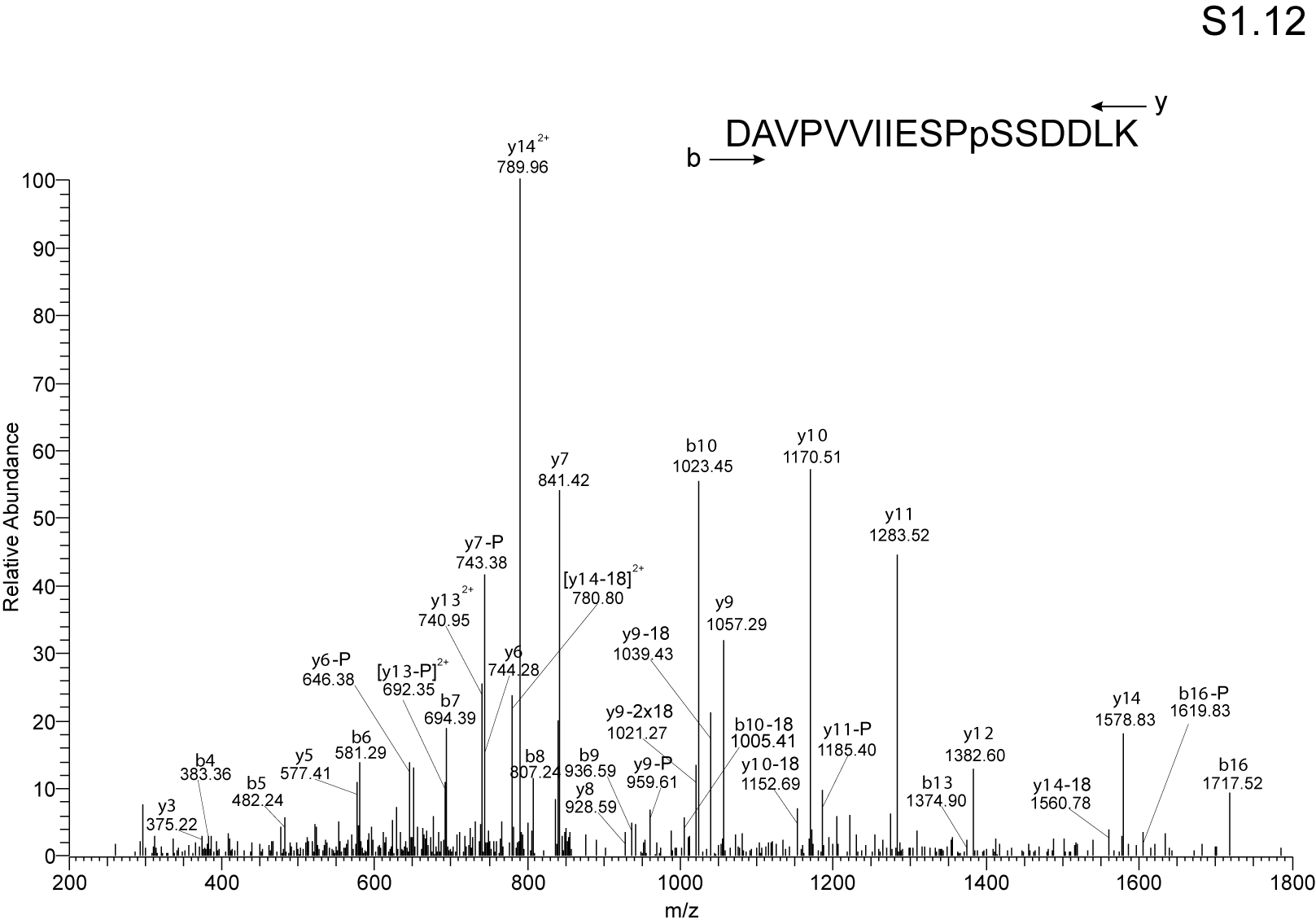


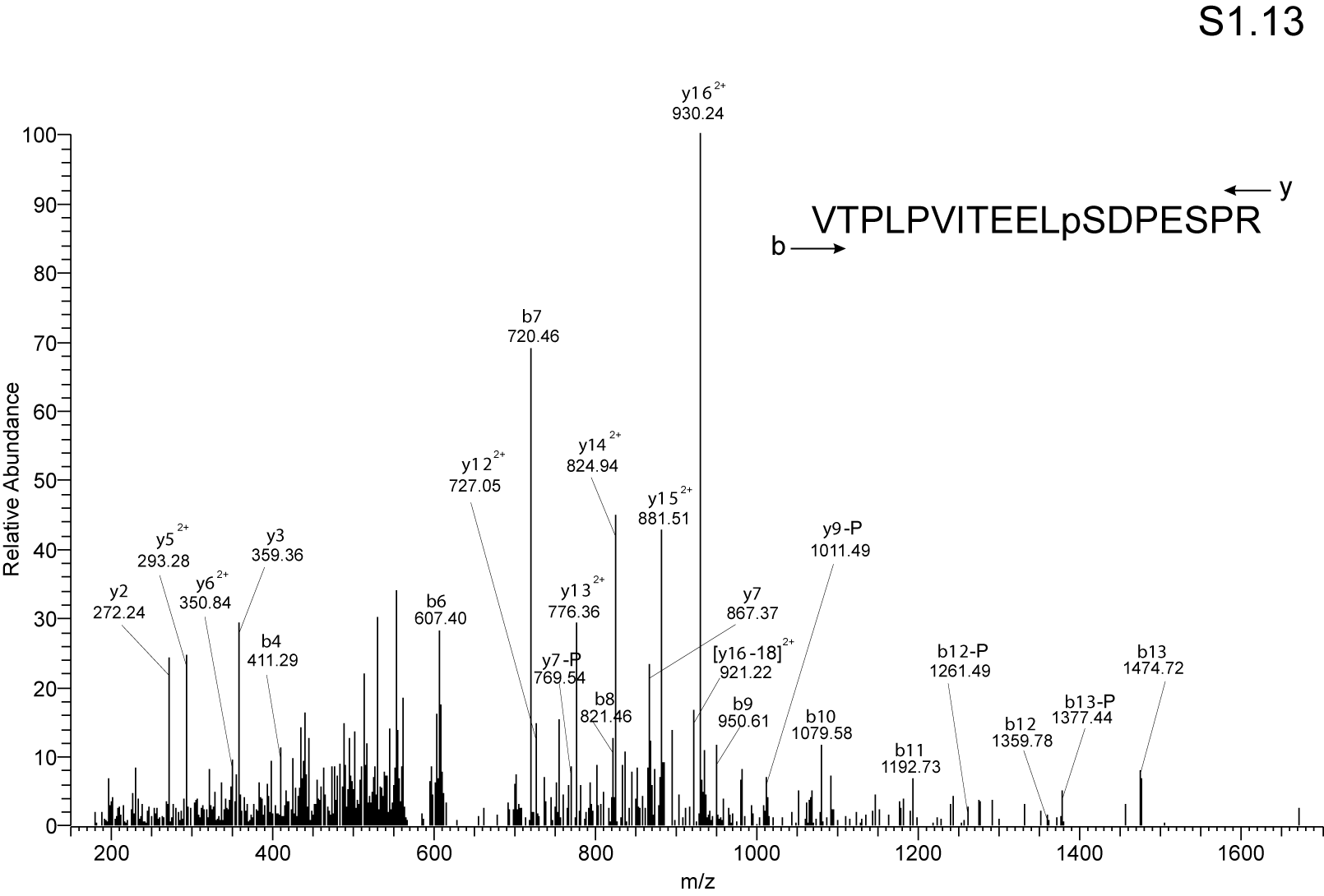


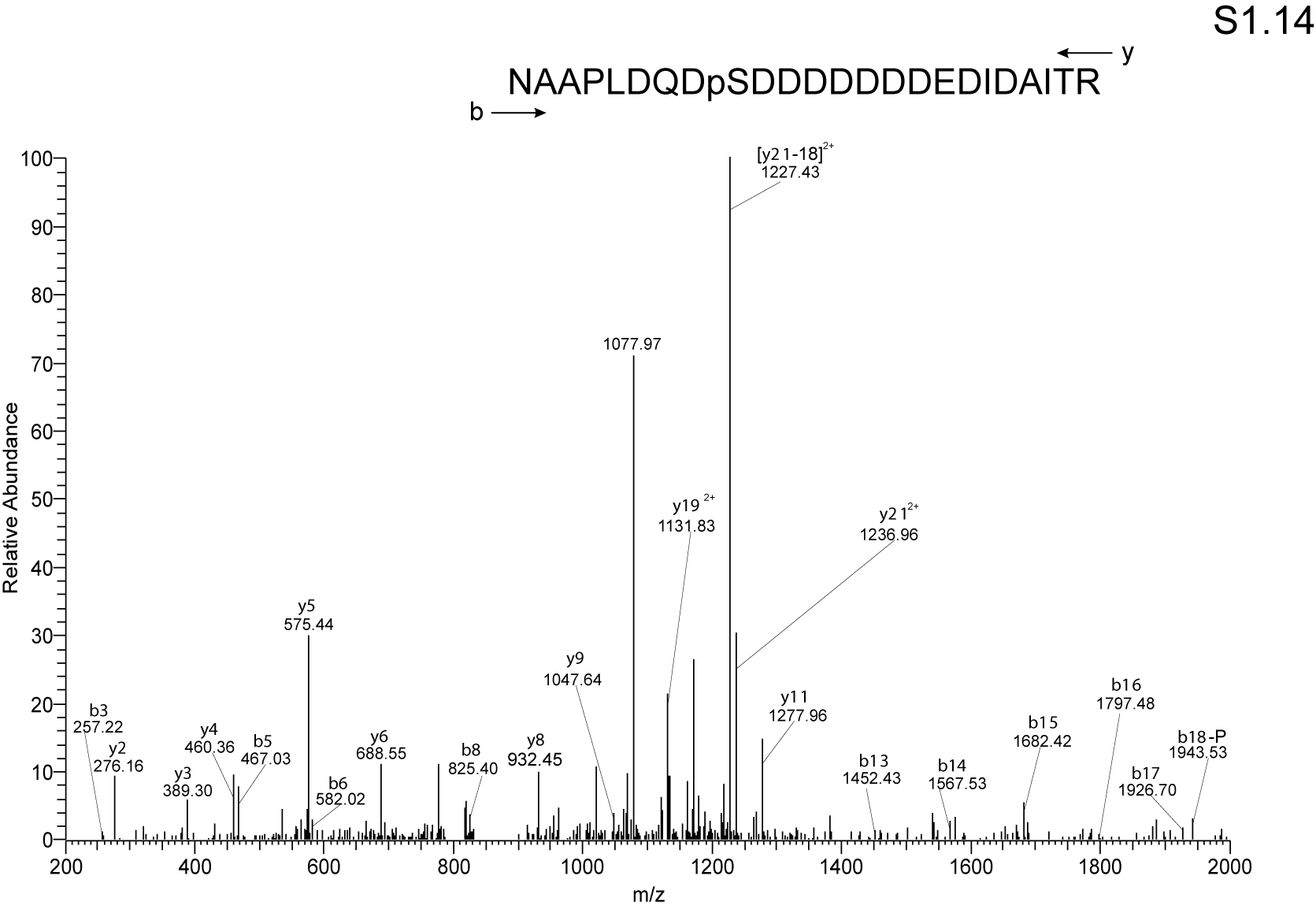


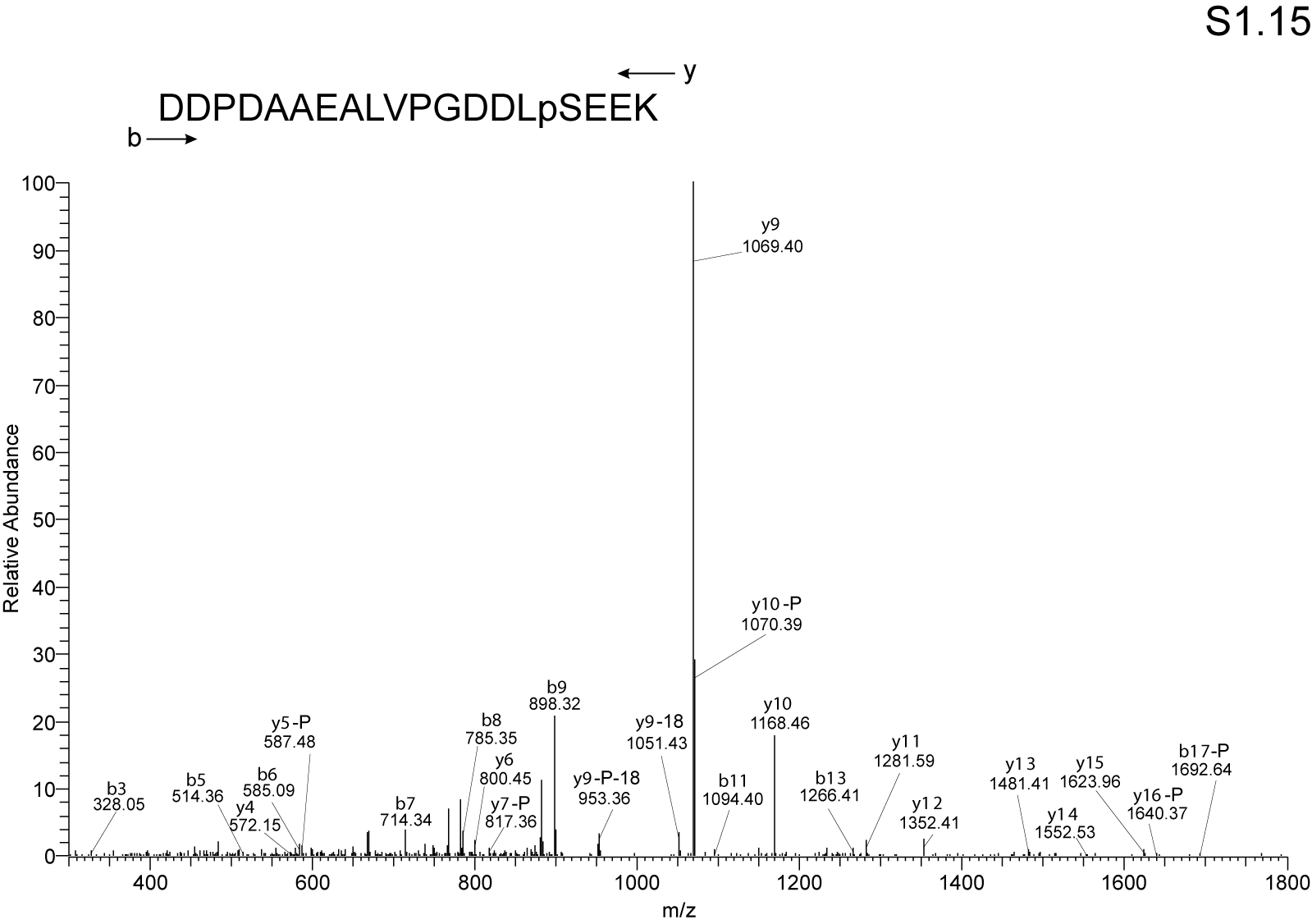


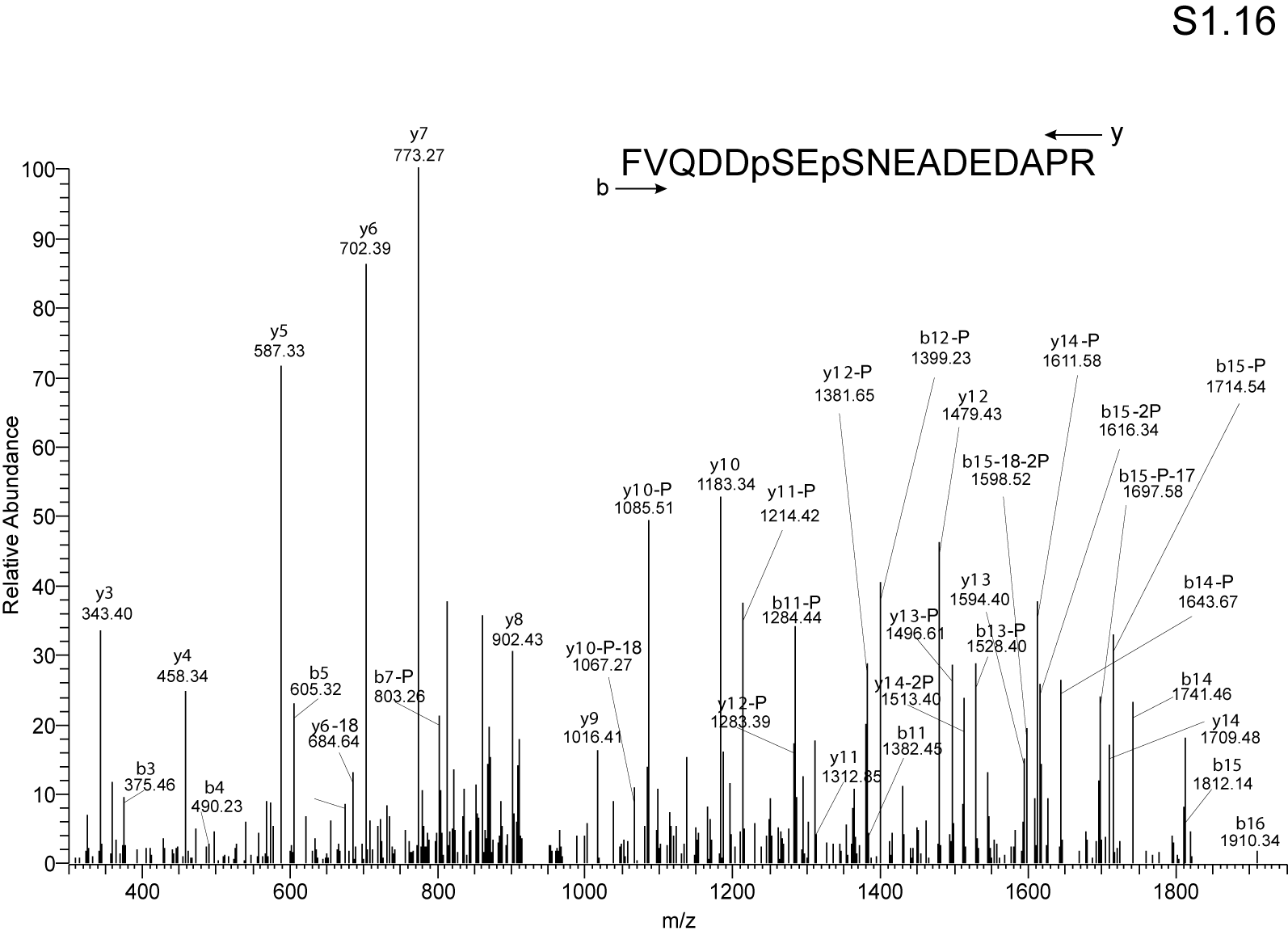


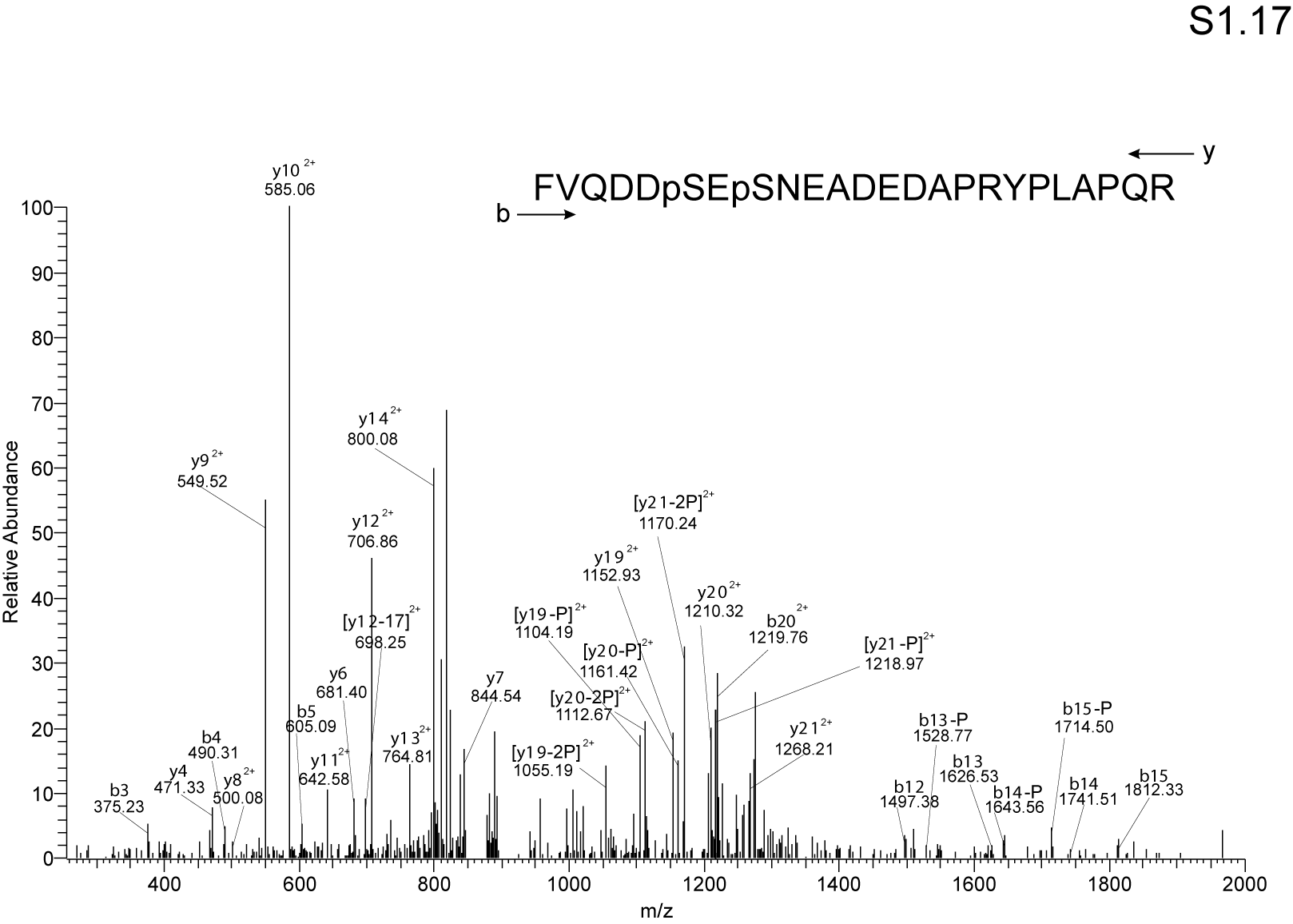


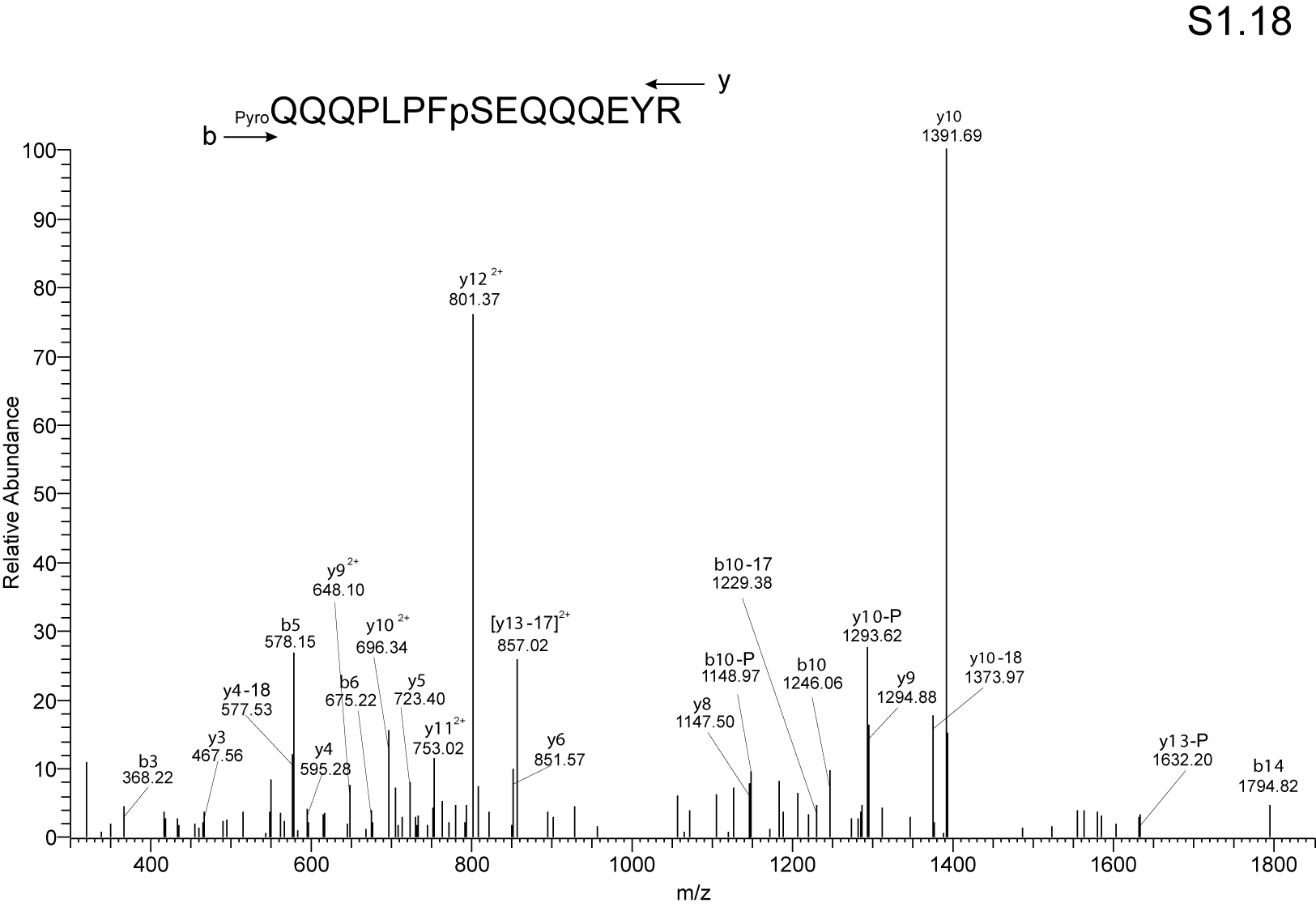


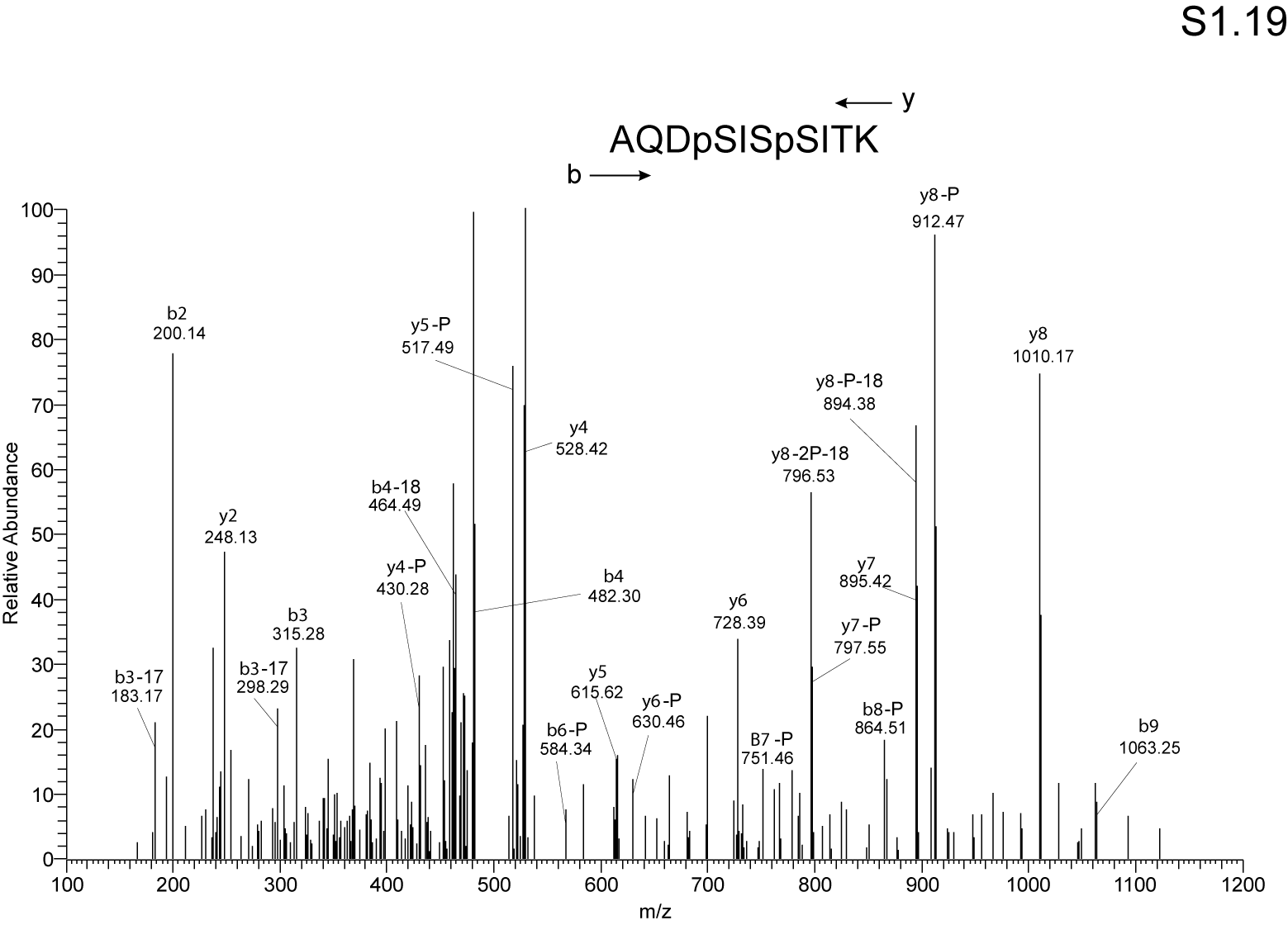


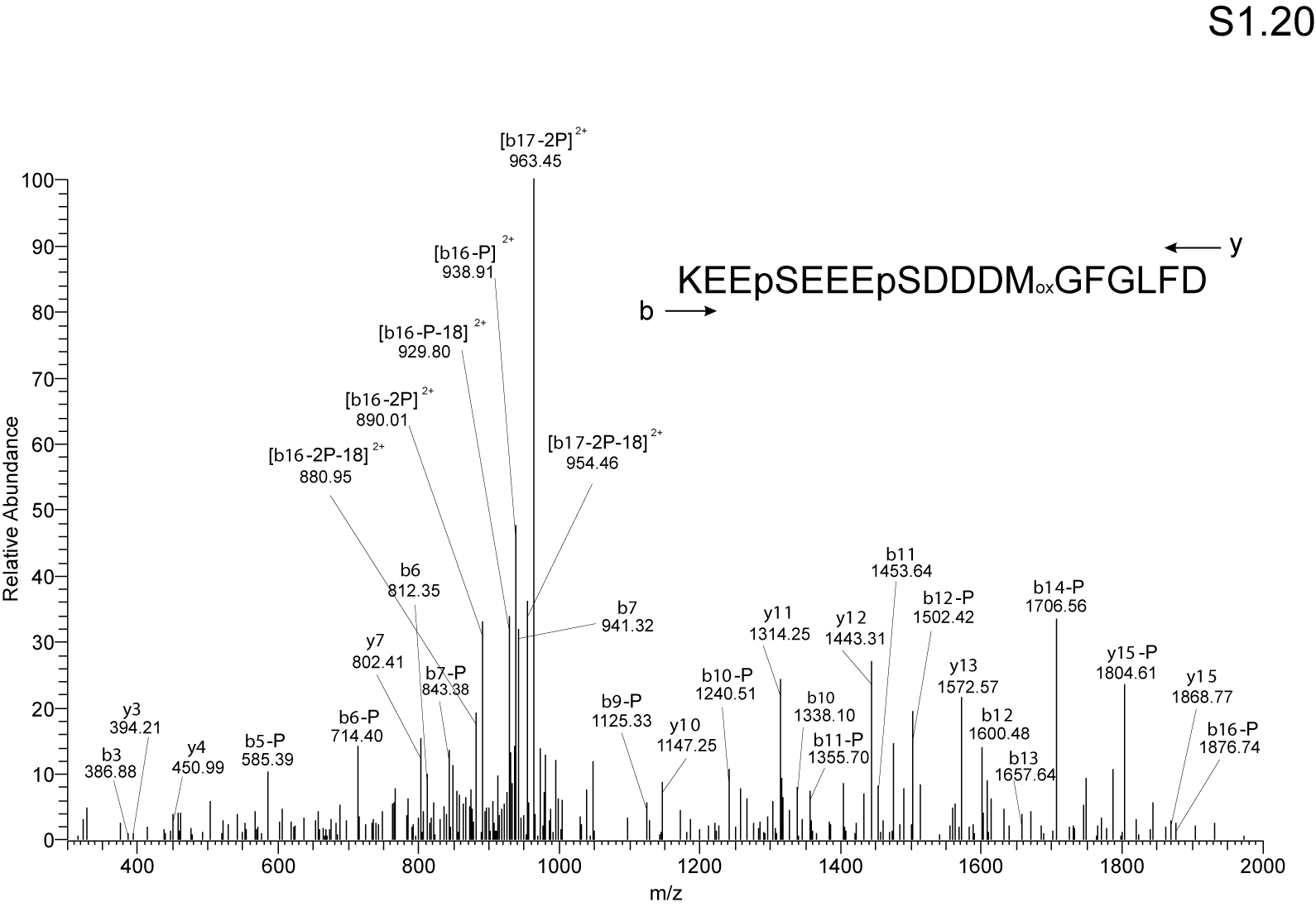


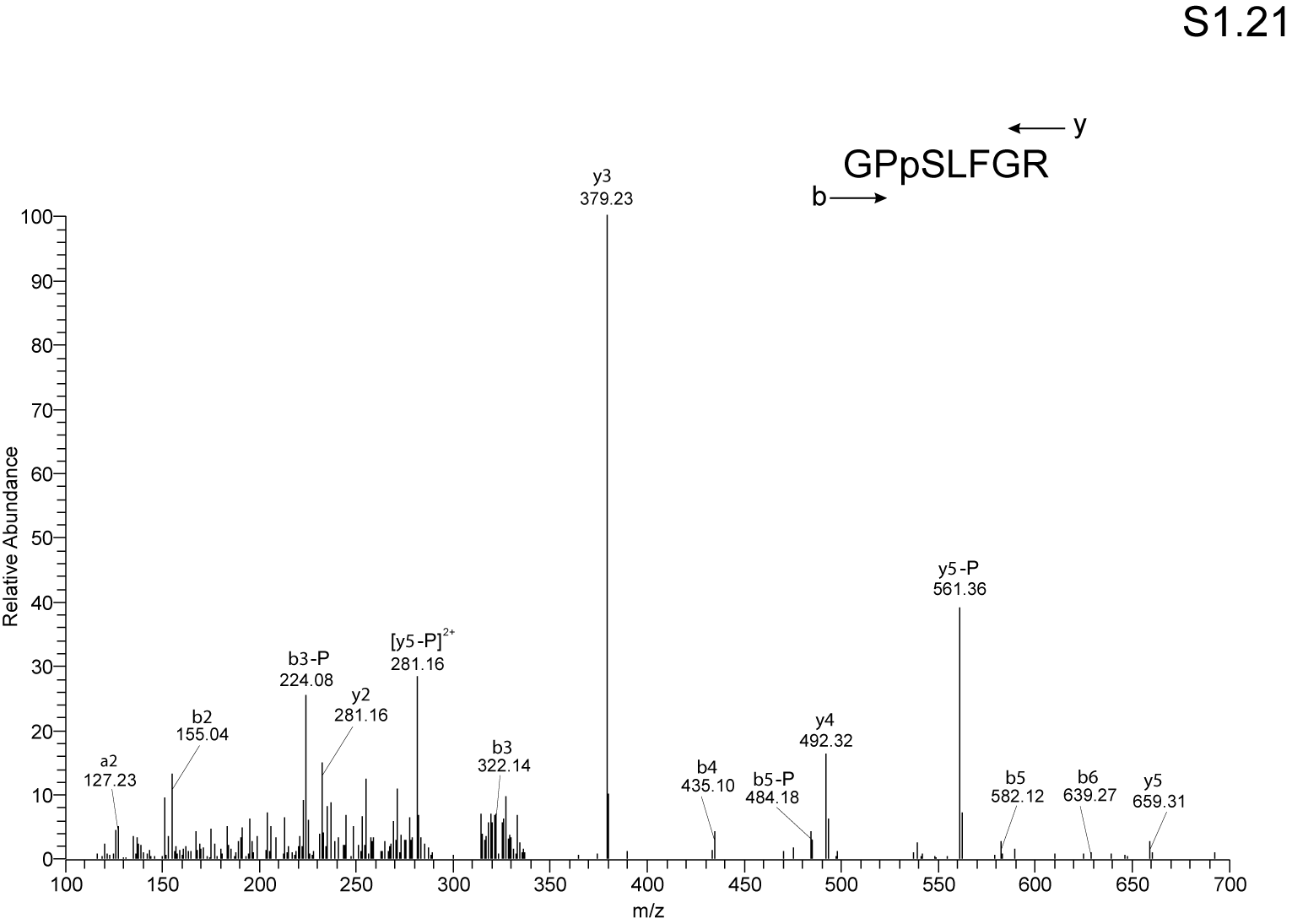


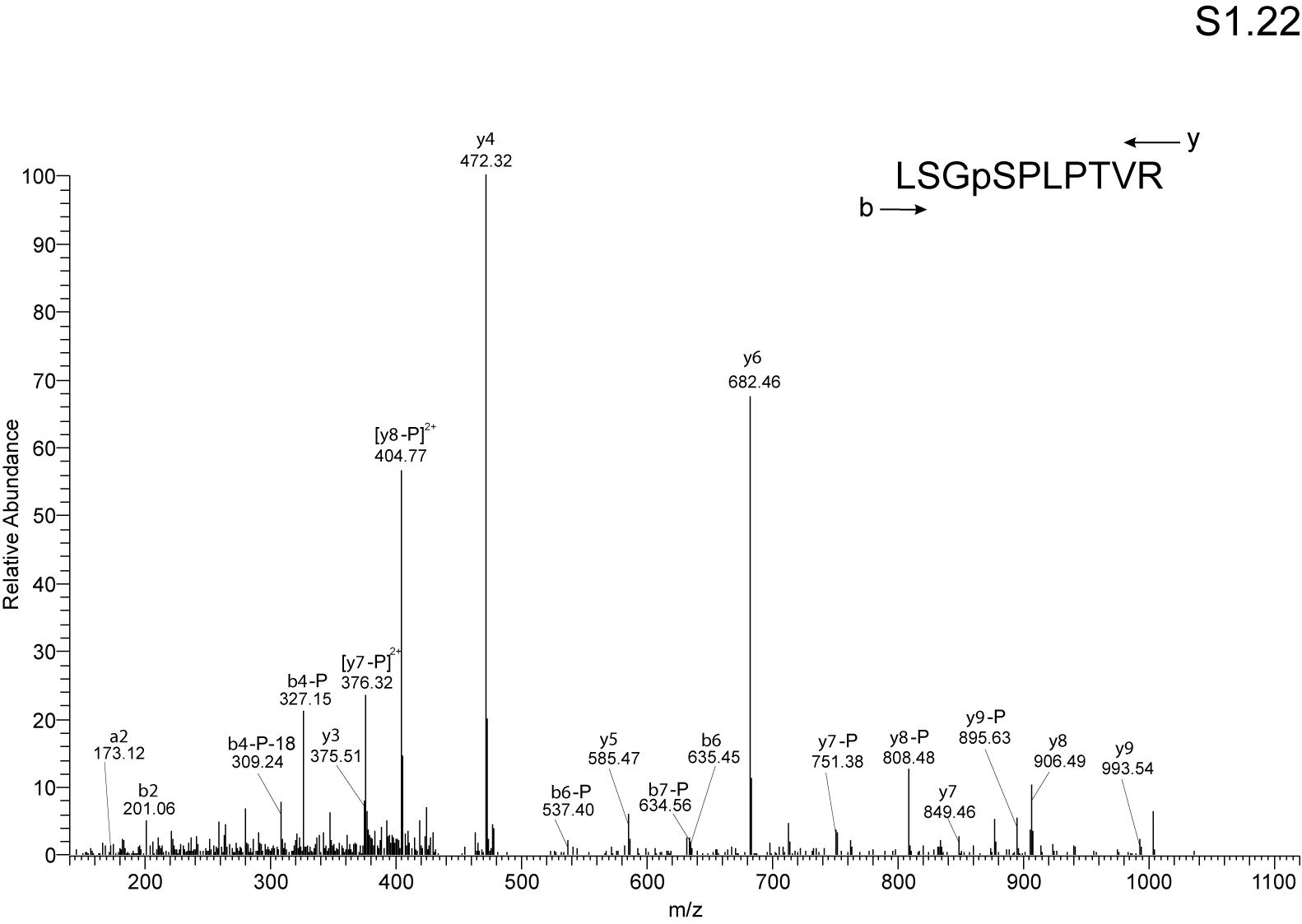


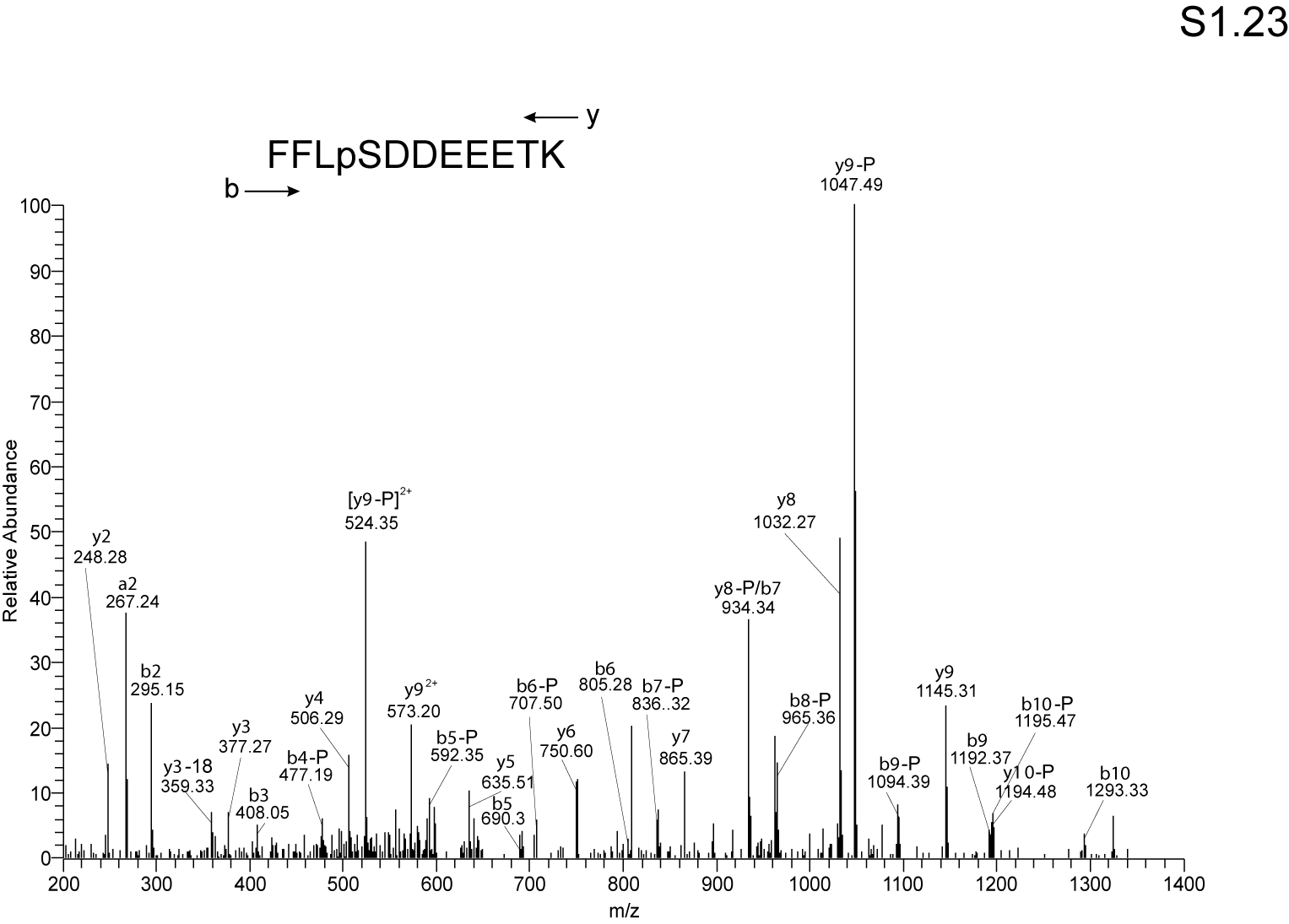


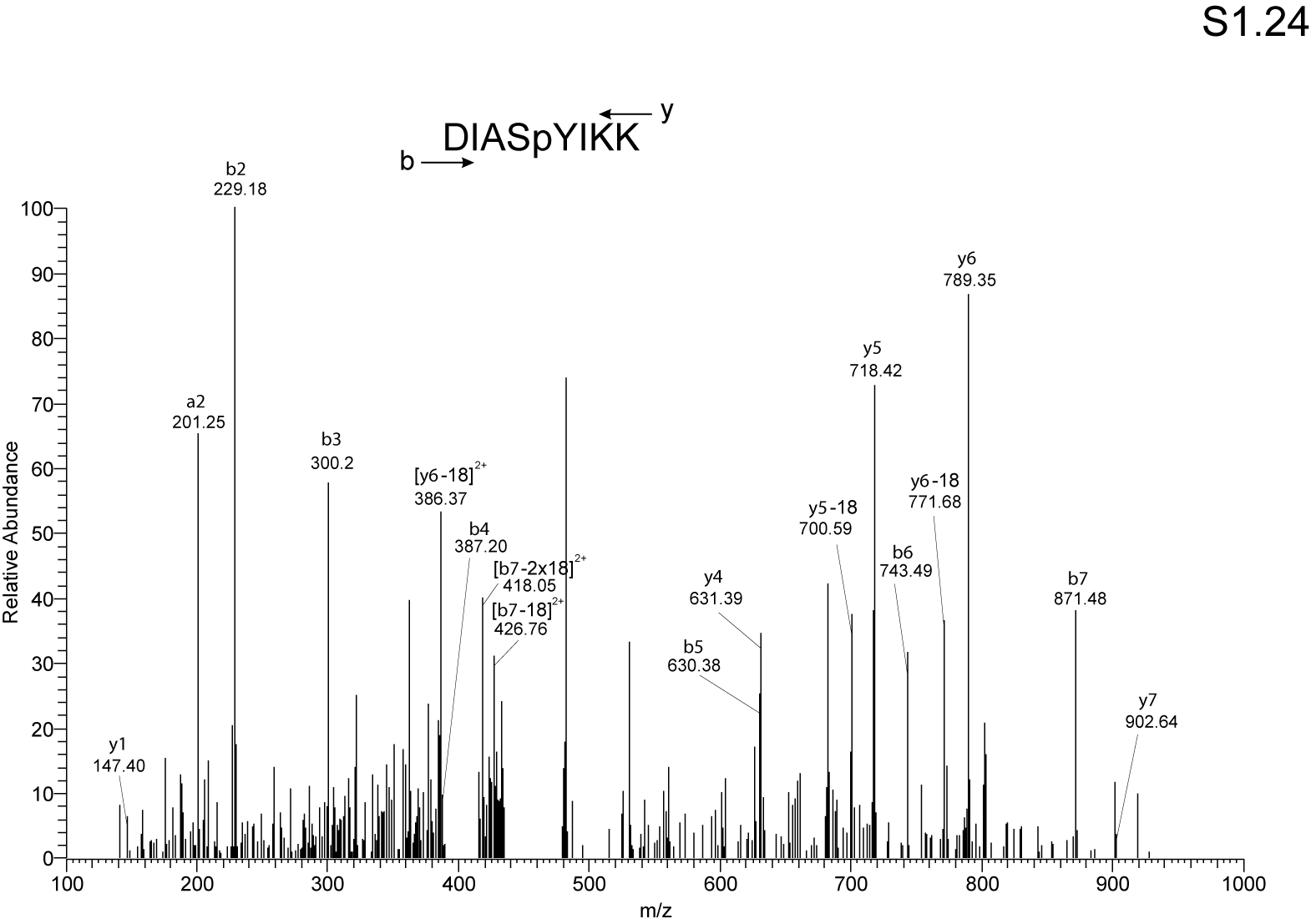


The spectra shown are the MaxQant-selected best identification spectra. Loss of phosphate, water (from Ser, Thr, Asp, or Glu) and ammonia (from Asn, Gln, Arg or Lys) is indicated as –P, -18 and -17, respectively. Numbering of spectra corresponds to numbering in Table 1.
